# The soluble cytoplasmic tail of CD45 (ct‐CD45) in human plasma contributes to keep T cells in a quiescent state

**DOI:** 10.1002/eji.201646405

**Published:** 2016-10-31

**Authors:** Alexander Puck, Stefan Hopf, Madhura Modak, Otto Majdic, Petra Cejka, Stephan Blüml, Klaus Schmetterer, Catharina Arnold‐Schrauf, Jens G. Gerwien, Klaus S. Frederiksen, Elisabeth Thell, Judith Leitner, Peter Steinberger, Regina Aigner, Maria Seyerl‐Jiresch, Gerhard J. Zlabinger, Johannes Stöckl

**Affiliations:** ^1^ Institute of ImmunologyCenter for PathophysiologyInfectiology and ImmunologyMedical University of ViennaViennaAustria; ^2^ Department for RheumatologyMedical University of ViennaViennaAustria; ^3^ Novo Nordisk A/SBiopharmaceuticals Research UnitMåløvDenmark; ^4^ Department for GynecologySt. Josef HospitalViennaAustria

**Keywords:** CD45, Immunoregulation, Quiescence, SLFN12, T‐cell activation

## Abstract

The cytoplasmic tail of CD45 (ct‐CD45) is proteolytically cleaved and released upon activation of human phagocytes. It acts on T cells as an inhibitory, cytokine‐like factor in vitro. Here, we show that ct‐CD45 is abundant in human peripheral blood plasma from healthy adults compared with plasma derived from umbilical cord blood and plasma from patients with rheumatoid arthritis or systemic lupus erythematosus. Plasma depleted of ct‐CD45 enhanced T‐cell proliferation, while addition of exogenous ct‐CD45 protein inhibited proliferation and reduced cytokine production of human T lymphocytes in response to TCR signaling. Inhibition of T‐cell proliferation by ct‐CD45 was overcome by costimulation via CD28. T‐cell activation in the presence of ct‐CD45 was associated with an upregulation of the quiescence factors Schlafen family member 12 (*SLFN12*) and Krueppel‐like factor 2 (*KLF2*) as well as of the cyclin‐dependent kinase (CDK) inhibitor *p27kip1*. In contrast, positive regulators of the cell cycle such as cyclin D2 and D3 as well as *CDK2* and *CDK4* were found to be downregulated in response to ct‐CD45. In summary, we demonstrate that ct‐CD45 is present in human plasma and sets the threshold of T‐cell activation.

## Introduction

Blood plasma is the liquid extracellular matrix of immune cells. It is a complex mixture of various components including nutrients such as electrolytes, lipids, sugars, and proteins 1, 2. According to the Human Plasma Proteome Reference Database, 1929 distinct proteins have been identified in human blood plasma so far including immune‐regulatory factors that could define immune cell functions. However, these immune regulatory factors and mechanisms by which they modulate immune cell function are incompletely characterized.

CD45 (PTPRC, leukocyte common antigen, B220, T200) is an abundant type I transmembrane protein expressed by all nucleated hematopoietic cells and their cellular precursors 3. The extracellular domain exists in various isoforms and shows little interspecies conservation, whereas, the intracellular domain has retained a high degree of conservation during mammalian evolution. CD45 is the prototypic receptor like protein tyrosine phosphatase and is a crucial regulator of antigen receptor signal transduction. Its enzymatic activity lies within the intracellular part, consisting of two tyrosine phosphatase homology domains, termed D1 and D2. While D1 displays phosphatase activity, D2 may serve to stabilize the former 4.

We have discovered an alternative function for CD45. Upon activation of human monocytes and granulocytes, CD45 is sequentially processed via serine/metalloproteinases and γ‐secretase. Cleavage by the latter yields a distinct fragment of 95 kDa comprising the cytoplasmic domains 5. Yurrita et al. recently confirmed this observation in MDCK cells 6. This soluble cytoplasmic tail of CD45 (ct‐CD45) was found to be released through activation‐induced cell death and to bind to a receptor expressed by activated primary human T lymphocytes, thereby transmitting an inhibitory signal to the cells 5. However, the physiological existence and relevance of ct‐CD45 has not been characterized.

Here, we investigated whether intact ct‐CD45 may be present as an inhibitory factor in human blood. In the present study, we established an ELISA specific for intact ct‐CD45 to confirm and quantify its presence in human plasma and serum samples. Furthermore, we analyzed the impact of ct‐CD45 on human T‐cell activation on a functional and molecular level. In summary, we demonstrate that ct‐CD45 is indeed a soluble factor in human plasma under steady‐state conditions, which contributes to the maintenance of T‐cell quiescence.

## Results

### ct‐CD45 is present in human plasma and modulates T‐cell function

**Figure 1:**
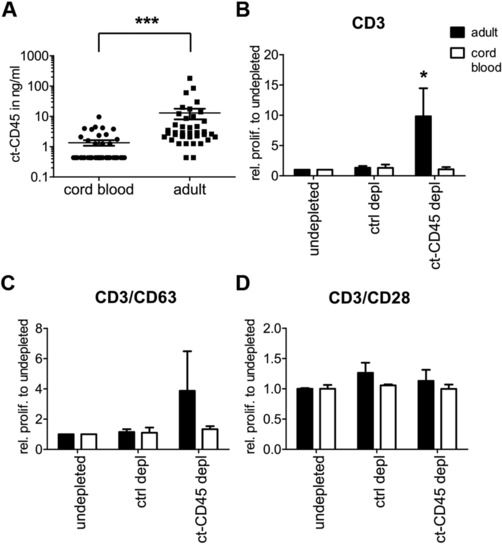
ct‐CD45 is present in human serum and modulates T‐cell function. (A) ct‐CD45 levels in human adult (*n* = 40) and cord blood plasma and serum (*n* = 41) were analyzed via ELISA. (B–D) Pooled adult or cord blood plasma was left untreated (undepleted), treated with bead‐coupled 8–301 mAb to deplete ct‐CD45 (ct‐CD45 depl) or was treated with bead‐coupled VIT200 mAb (ctrl depl) as a control. These plasma samples were then used in proliferation assays of CD3^+^ T cells that were activated for 4 days via plate‐bound (B) CD3, (C) CD3/CD63, or (D) CD3/CD28 antibodies. (B–D) Proliferation was measured on day 3 via thymidine incorporation. Data are displayed relative to the proliferation of cells treated with undepleted plasma (rel. prolif. to undepleted) and are pooled from two independent experiments with two to three samples per experiment (*n* = 5). Results are displayed as mean ± SEM. *p* values: *p < 0.05; **p < 0.01; ***p < 0.001. (A) Mann–Whitney *U* test or (B–D) Kruskal–Wallis with Dunn's posttest (multiple comparisons) was used. Only significant differences are indicated.

Activation of human phagocytes isolated from peripheral blood can lead to generation of ct‐CD45 5. Since proteome analyses of human plasma have revealed that CD45‐derived peptides are present in plasma, an ELISA was established to detect intact ct‐CD45 consisting of D1 and D2, but not D1 or D2 alone (Supporting Information Fig. 1A). The ELISA specifically detected ct‐CD45, which was confirmed by exchanging the ct‐CD45‐specific mAb 8–301 5 with mAb VIT200, which recognizes the extracellular domain of CD45 (data not shown). A screen using this system showed that ct‐CD45 is detectable in both, adult and umbilical cord blood plasma or serum (Fig. 1A). However, while the mean concentration in cord blood plasma (1.36 ± 0.28 ng/mL) was close to the detection limit of the ELISA (0.8 ng/mL), ct‐CD45 levels in adult plasma were substantially higher (12.98 ± 5.04 ng/mL) (Fig. 1A). ct‐CD45 (4.6 ng/mL) was also found in Octaplas, a commercial, solvent/detergent‐treated plasma preparation pooled from several thousand donors. Total levels of ct‐CD45 were comparable between serum and plasma pools from either adult or cord blood (Supporting Information Fig. 1B).

Since ct‐CD45 levels were different between adult and cord blood, we hypothesized that these levels might change with age in later life. However, linear regression analysis did not reveal any significant correlation of ct‐CD45 with age (Supporting Information Fig. 2A) and did not show any difference between the male and female study population (Supporting Information Fig. 2B).

To test whether physiological levels of ct‐CD45 have an impact on human T‐lymphocyte activation, ct‐CD45 was depleted via immunoprecipation from human plasma (Supporting Information Fig. 1C and D) and these samples were then used in T‐cell proliferation assays (Fig. 1B–D). In this setting, adult plasma depleted of ct‐CD45 elicited stronger proliferative responses of human T cells than undepleted or control‐depleted plasma. This was predominantly observed when T cells where activated via CD3 mAbs alone (Fig. 1B) and was less pronounced or absent when costimulated via CD63 (Fig. 1C) or CD28 (Fig. 1D). Conversely, ct‐CD45 levels in cord blood plasma appeared to be too low to have any functional impact on T‐cell proliferation (Fig. 1B–D), since no ct‐CD45 could be immunoprecipitated from plasma derived from human cord blood (Supporting Information Fig. 1D).

### ct‐CD45 is an inhibitor of T‐cell function in the absence of sufficient costimulation

To further explore the impact of ct‐CD45 on T‐cell function, T cells were activated via plate‐bound CD3, CD3/CD63, or CD3/CD28 in the presence of immobilized ct‐CD45‐Ig or CTLA4‐Ig as a control fusion protein. In this setting, ct‐CD45‐Ig (hence referred to as ct‐CD45) was used at saturating conditions (Supporting Information Fig. 3 and 5). CTLA4‐Ig has been described to inhibit the T‐cell antigen‐presenting cell interaction, but does not act directly on T cells 7, 8, thus serving as negative control in this system.

**Figure 2:**
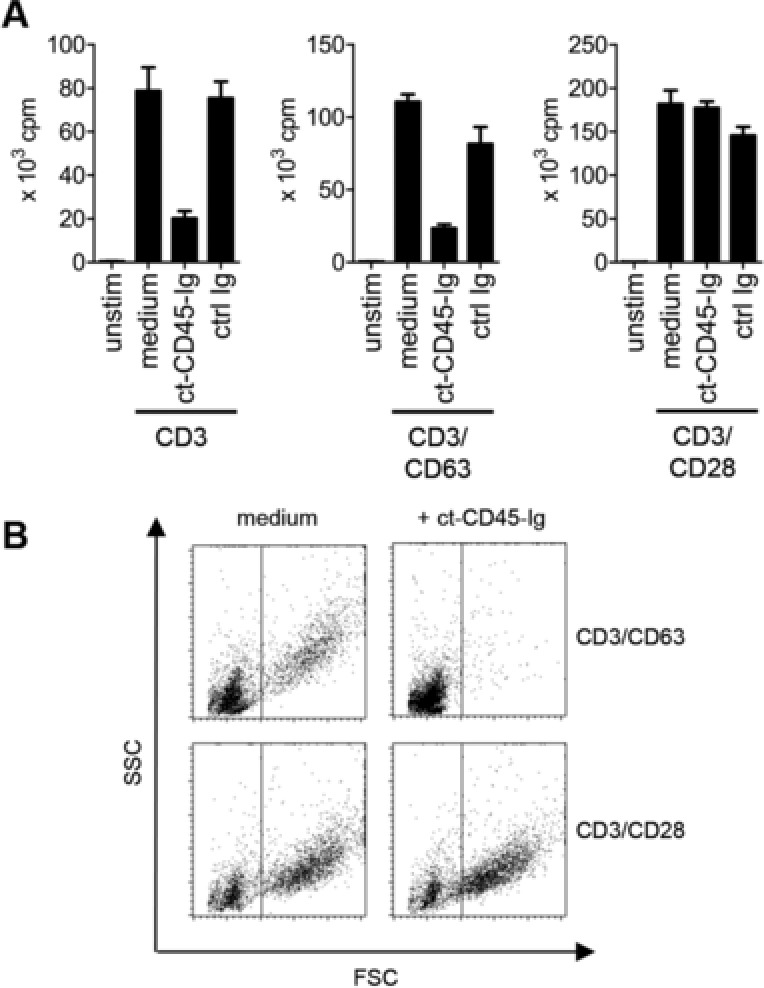
ct‐CD45 preferentially inhibits the activation of suboptimally stimulated T cells. (A) Proliferation of human CD3^+^ T cells that had been activated for 4 days via plate‐bound CD3, CD3/CD63, or CD3/CD28 antibodies in the presence of medium alone, ct‐CD45‐Ig or control fusion protein (ctrl Ig) was analyzed via thymidine incorporation on day 3. cpm, counts per minute. unstim., unstimulated control. (B) forward scatter (FSC) and side scatter (SSC) profiles of T cells that had been stimulated as indicated above, were analyzed via flow cytometry. (A) Data are displayed as mean ± SD of triplicate measurements and (A, B) are representative of at least three independent experiments.

T‐cell growth was impaired in the presence of ct‐CD45 not only for T cells activated via CD3 but also for T cells costimulated via CD63, whereas CD28‐mediated costimulation overcame the inhibitory effect (Fig. 2A). The inhibition of T‐cell proliferation was not restricted to CD4^+^ (Supporting Information Fig. 4A) or CD8^+^ T cells (Supporting Information Fig. 4B), since similar results were observed for both T‐cell subsets. Interestingly, ct‐CD45 did not show any impact on the proliferation of naïve, cord blood T cells (Supporting Information Fig. 4C), indicating that responsiveness to this inhibitory signal may be restricted to adult T cells.

**Figure 3:**
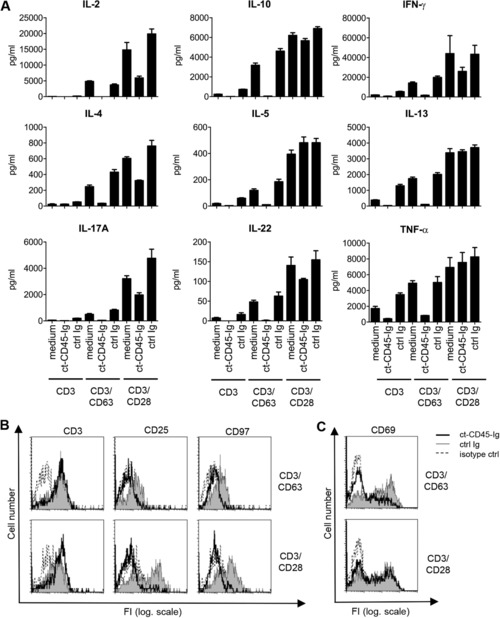
ct‐CD45 modulates the release of T‐cell cytokines and activation markers. (A) Cell culture supernatants obtained from human CD3^+^ T cells that were activated for 4 days via plate‐bound CD3, CD3/CD63 or CD3/CD28 antibodies in the presence of medium alone, ct‐CD45‐Ig or control Ig fusion protein (ctrl Ig) were analyzed for the indicated cytokines via Luminex. (B, C) T‐cell activation markers of cells that had been activated via CD3/CD63 and CD3/CD28 antibodies as indicated above were analyzed via flow cytometry. Histograms with thick black lines represent staining of cells that were activated in the presence of ct‐CD45‐Ig. Grey, filled histograms are ctrl Ig‐treated cells, dotted line is the isotype control staining. FI, fluorescence intensity; log. scale, logarithmic scale. (A) Data are displayed as mean ± SD of triplicate measurements and (A, B) are representative of three independent experiments.

T lymphoblast formation was unaffected in adult T cells stimulated via CD3/CD28, while CD3/CD63/ct‐CD45‐stimulated cells remained rather small, resembling resting T cells (Fig. 2B, Supporting Information Fig. 4D). Cytokine release (IL‐2, IFN‐γ, IL‐4, IL‐5, IL‐13, IL‐10, IL‐17A, IL‐22, and TNF‐α) was inhibited for CD63, whereas CD28 costimulated T cells were only inhibited in the production of IL‐2, IL‐4, IL‐17, and IL‐22 (Fig. 3A). Classical T‐cell activation markers (Fig. 3B) were reduced for both types of costimulation, with the exception of CD69, which only was slightly affected (Fig. 3C). Similarly, cell surface expression and modulation of the TCR/CD3 complex upon T‐cell activation was not differentially regulated by ct‐CD45 (Fig. 3B).

### ct‐CD45 promotes the expression of quiescence genes

Since T cells that were activated in the presence of ct‐CD45 were strongly inhibited in their function, we aimed to characterize the underlying molecular mechanism. Thus, global gene expression analysis was performed, comparing primary human T cells activated via CD3/CD63 or CD3/CD28 in the presence or absence of ct‐CD45‐Ig.

**Figure 4:**
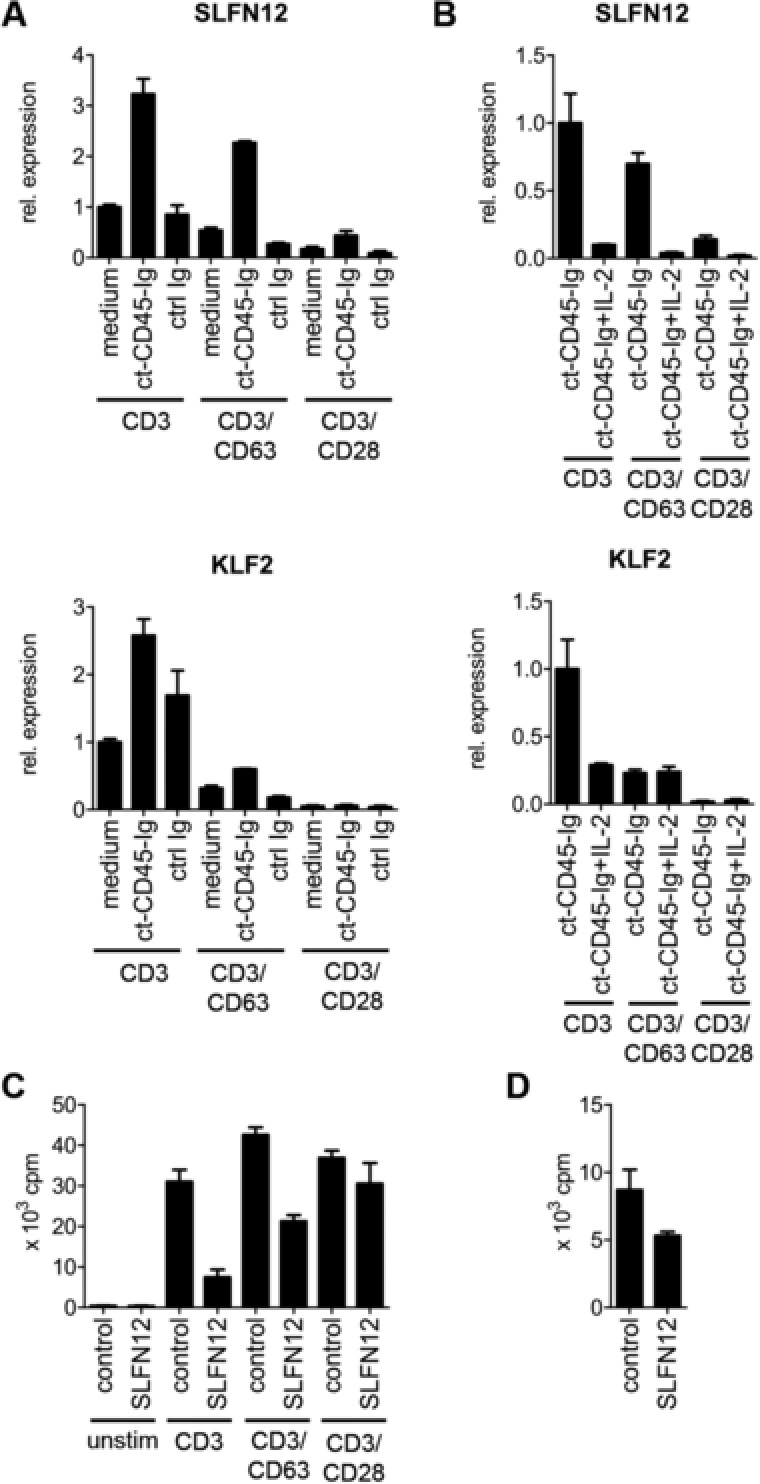
Upregulation of T‐cell quiescence factors in T cells activated in the presence of ct‐CD45. Human CD3^+^ T cells were stimulated via plate‐bound CD3, CD3/CD63, or CD3/CD28 antibodies in the presence of medium alone, ct‐CD45‐Ig, or ctrl Ig. Cells were harvested after 48 h. (A) mRNA levels of *KLF2* and *SLFN12* were analyzed via qPCR relative to *GAPDH*. (B) T cells were activated for 48 h in the presence of ct‐CD45‐Ig with or without recombinant IL‐2 (10 U/mL). *SLFN12* and *KLF2* expression was analyzed via qPCR. (C) CD3^+^ T cells were retrovirally transduced with *SLFN12* or empty control vector and where restimulated via indicated stimuli for 4 days. T‐cell proliferation was measured via thymidine incorporation on day 3. (D) Jurkat T cells were transduced with *SLFN12*. Proliferation of the cell line was determined on day 4 after gene transduction measuring thymidine incorporation for another 18 h. cpm, counts per minute. (A–D) Data are displayed as mean ± SD and are representative of at least three independent experiments.

Inhibition of T‐cell activation is typically associated with expression of inhibitory factors, such as *CB*L‐b 9, EG*R3* 10, *Tob1* 11, or *DGK‐*α 12. However, none of these genes were found to be overexpressed in ct‐CD45‐treated cells as compared to controls (Supporting Information Table 1). Instead, two genes were found to be upregulated—Schlafen family member 12 (*SLFN12*) and Krueppel‐like factor 2 (*KLF2*) (Supporting Information Table 1, Fig. 4A). While the zinc finger transcription factor KLF2 has been implicated in the induction and maintenance of T‐cell quiescence before 13, 14, little is known about SLFN12 15.

Expression analysis of both factors during human T‐cell activation in the presence of ct‐CD45 revealed upregulation of SLFN12 6 h after stimulation, while KLF2 was not differentially expressed at this early time point (Supporting Information Fig. 6). However, both genes were downregulated following stimulation in the absence of ct‐CD45 (Supporting Information Fig. 7A and 16), indicating a possible function in the regulation of T‐cell activation. Since the T‐cell growth factor IL‐2 plays an important role in cell‐cycle entry and progression of T cells 17, we hypothesized, that addition of IL‐2 might modulate the expression of both factors. Indeed, exogenous IL‐2 was able to strongly downregulate *SLFN12* mRNA in ct‐CD45‐treated T cells in all activation conditions tested (Fig. 4B), while *KLF2* expression could only be modulated in the absence of costimulation. Since the role of KLF2 as a negative regulator of T‐cell growth has been well established 13, 14, we focused on the characterization of *SLFN12* in human T‐cell function. Various approaches were undertaken in an attempt to knockdown *SLFN12* in primary T cells including transfection of synthetic siRNA via lipofection or nucleofection and via retroviral shRNA, but no significant knockdown was achieved (data not shown).

Thus, another approach was applied, in which *SLFN12* was overexpressed in primary T cells via retroviral gene transduction (Supporting Information Fig. 7B). Expression of the transgene inhibited the proliferation of T cells stimulated via CD3 or CD3/CD63, while showing hardly any impact on CD3/CD28 stimulation (Fig 4C). Similarly, *SLFN12* overexpression in the continuously proliferating T‐cell leukemia cell line Jurkat (Supporting Information Fig. 7C) led to a reduction in overall growth rates (Fig. 4D) and reduced cellular viability (Supporting Information Fig. 7D). *SLFN12* was also overexpressed in the nonhematopoietic cell line Hela Ohio, which died rapidly within 3 days after gene transduction, while control‐transduced cells could be maintained normally (Supporting Information Fig. 7E), indicating that *SLFN12*‐mediated inhibition of cell growth might not be restricted to T lymphocytes.

**Figure 5:**
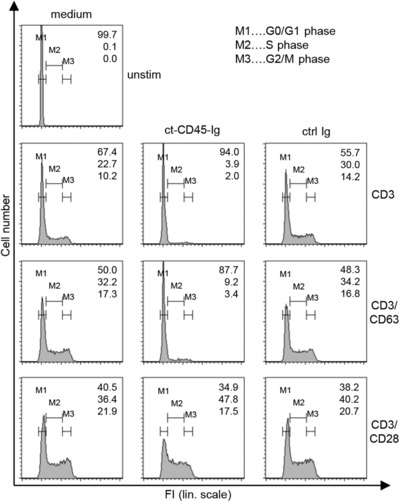
ct‐CD45 induces a G_0_/G_1_ cell‐cycle arrest in T cells. T cells were stimulated for 4 days via plate‐bound CD3, CD3/CD63, or CD3/CD28 antibodies in the presence of medium alone, ct‐CD45‐Ig, or ctrl Ig. Cells were then harvested, fixed, and stained with PI following analysis of single cells via flow cytometry. FI, fluorescence intensity; lin. scale, linear scale; unstim, unstimulated control. Numbers given indicate percentages of cells in the respective phase of the cell cycle. Data are representative of two independent experiments.

**Figure 6:**
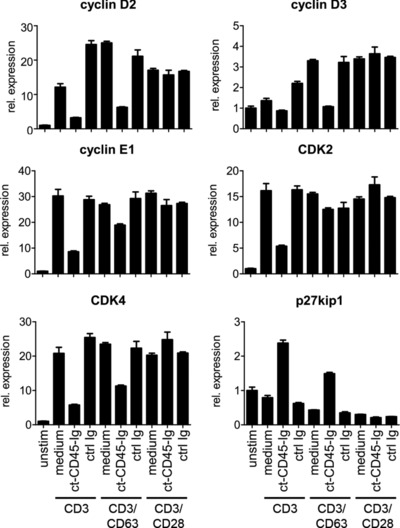
ct‐CD45 affects the expression of cell‐cycle regulatory factors. T cells were activated via plate‐bound CD3, CD3/CD63, or CD3/CD28 antibodies in the presence of ct‐CD45‐Ig or ctrl Ig for 4 days. Cells were then harvested and subjected to qPCR analysis measuring the expression of the indicated cell‐cycle regulatory factors. *CD3E* was used as a reference gene. rel. expression, relative expression; unstim, unstimulated control. Data are shown as mean ± SD of triplicates and are from a single experiment representative of two independent experiments.

The increased expression of quiescence genes as well as decreased blastogenesis suggested an early cell‐cycle arrest of T cells activated in the presence of ct‐CD45. Therefore, cell‐cycle distribution of these cells was analyzed. Indeed, T cells stimulated with CD3/ct‐CD45 or CD3/CD63/ct‐CD45 hardly entered S phase and mostly remained in the G_0_/G_1_ phase. Conversely, CD3/CD28/ct‐CD45 stimulated cells displayed similar cycling compared to control‐stimulated cells (Fig. 5). Gene expression analysis of the G_1_ cyclins D2, D3, and E1 and of cyclin‐dependent kinases *CDK2* and *CDK4* found reduced expression in CD3/ct‐CD45, but also, to a minor degree, in CD3/CD63/ct‐CD45‐activated cells (Fig. 6). Conversely, the CDK inhibitor *p27kip1* was found to be upregulated in both conditions (Fig. 6). Again, CD3/CD28 activated cells were not affected by ct‐CD45 treatment and showed similar expression of cell‐cycle regulatory factors to controls.

### Reduction of SLFN12 and ct‐CD45 levels in rheumatoid arthritis and systemic lupus erythematosus patients

**Figure 7:**
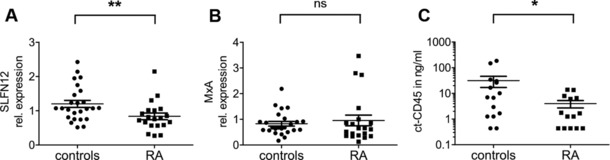
SLFN12 and ct‐CD45 are downregulated in rheumatoid arthritis patients. Primary CD3^+^ T cells were isolated from the peripheral blood of healthy controls and patients with rheumatoid arthritis (RA). (A) *SLFN12* and (B) *MxA* mRNA expression was measured in purified cells via qPCR using *B2M* as a reference gene (controls *n* = 25, patients *n* = 20). (C) ct‐CD45 levels were analyzed in plasma of healthy controls and RA patients (controls *n* = 15, patients *n* = 14) via ELISA. Data are displayed as mean ± SEM. *p* values: *p < 0.05; **p < 0.01. Mann–Whitney *U* test.

To investigate whether these findings bear significance for human disease, T cells were purified from the peripheral blood of individuals diagnosed with rheumatoid arthritis (RA). RA been characterized by the presence of activated T cells in the peripheral blood 18, 19, 20 and has also been associated with the expression of a type I interferon signature in a subset of RA patients 21. Like other members of the *SLFN* family, *SLFN12* is inducible by type I interferons 16, 22. According to the hypothesis that *SLFN12* expression could be modulated by both signals in these patients, we analyzed its expression with respect to healthy controls. Strikingly, RA patients showed a significant reduction of *SLFN12* mRNA (Fig. 7A), indicating that signals leading to T‐cell activation might overcome type I interferon signaling in this setting. As a control, expression of the interferon‐inducible gene *MxA* was determined. As expected, *MxA* was upregulated at least in some patients, albeit the differences found in the total study population did not reach statistical significance (Fig. 7B).

Analysis of ct‐CD45 expression in patient plasma revealed lower levels compared to healthy controls (Fig. 7C), suggesting that reduced ct‐CD45 levels might facilitate or reflect the development of aberrant immune response in these patients. To test whether ct‐CD45 levels were directly linked to clinical parameters of RA patients, we performed linear regression analysis with levels of C‐reactive protein (Supporting Information Fig. 8A) and rheuma factor (Supporting Information Fig. 8B) as well as with the disease activity scores CDAI and DAS28 (Supporting Information Fig. 8C and D), but found no statistically significant correlation.

We also analyzed ct‐CD45 levels in the plasma of patients diagnosed with systemic lupus erythematosus (SLE) and found significant reduction of ct‐CD45 levels (Supporting Information Fig. 9). Since leukopenia may occur in SLE patients 23, we analyzed whether there was a link between leukocyte numbers and ct‐CD45 levels, but found no significant correlation (Supporting Information Fig. 10). Most individuals of the study population were under treatment with anti‐inflammatory drugs (Table 1), suggesting that ct‐CD45 levels might be altered due to immunosuppressive treatment. A large proportion of RA patients received methotrexate as a standard immunosuppressant 24, thus we decided to compare individuals that received methotrexate to patients that were treated with other drugs. Interestingly, a strong tendency for lower ct‐CD45 levels was observed in RA patients treated with methotrexate compared to subjects with other treatments (Supporting Information Fig. 11A). An even more distinct difference indicating effects of certain immunosuppressive drugs on ct‐CD45 levels was obtained for SLE patients, which were grouped according to chloroquine and azathioprine treatment. In this context, a statistically significant difference was observed between the two groups, since ct‐CD45 was nearly absent in the azathioprine‐treated group (Supporting Information Fig. 11B).

**Table 1:** Patient characteristics

Symbol	RA (*n* = 26)	SLE (*n* = 8)
Age (years)	63.0 ± 10.0	41.3 ± 12.6
Female sex (*n*, %)	17 (65.4)	8 (100)
Disease duration	12.9 ± 7.9	8.7 ± 7.2
Treatment (*n*)		
TNF inhibitors	8	0
Methotrexate (folic acid antagonist)	12	1
CTLA‐4‐Ig	2	0
Leflunomide (DHODH inhibitor)	5	0
Chloroquine	1	6
Azathioprine (purine analogue)	0	3
Other treatments	0	1
Rheuma factor (IU/mL)	170 ± 231	N/A
C‐reactive protein (mg/L)	0.7 ± 0.8	N/A
Disease activity scores		
CDAI	12.0 ± 9.5	N/A
DAS28	3.6 ± 1.4	N/A
Leukocyte numbers (×10^6^/mL)	N/A	4.7 ± 1.9 × 10^6^/mL

Clinical parameters of patients involved in this study. Age and clinical parameters are given as mean ± SD.

DHODH: dihydroorotate dehydrogenase; DAS: disease activity score; CDAI: clinical disease activity index.

## Discussion

T cells circulate in peripheral blood and lymphoid organs in a resting or quiescent state that is characterized by small cellular size and low metabolic rate. T‐cell quiescence is not a default pathway that persists until a T cell is activated by binding of its cognate antigen to the TCR, but is maintained under active transcriptional control ensuring the expression of a quiescent gene program 25, 26. Maintenance of T‐cell homeostasis is important to prevent autoimmunity. Extrinsic factors that regulate the quiescent state in human T cells are largely unknown. Here, we demonstrate that ct‐CD45 is present in human plasma and acts as a regulatory factor to keep T cells in a resting state.

Human plasma is the multifunctional liquid matrix of immune cells. It consists of different types of molecules including hundreds of different proteins 1, 2. ct‐CD45 is one of many potential immune regulatory factors in plasma. CD45 is listed by the Human Plasma Proteome Reference Database as one of 1929 canonical proteins found in human plasma 27, 28. Closer analysis of the MS data showed that three of four identified peptides originate from ct‐CD45, thus supporting our finding that ct‐CD45 is present in plasma.

We show in this study, that ct‐CD45 acts as a potent inhibitor of T‐cell activation via the TCR in the absence of strong costimulation. This functional behavior of ct‐CD45 supports the concept that ct‐CD45 is not proapoptotic or a global inhibitory molecule but a regulatory factor that sets the threshold to prevent undesired activation of T cells. This effect was observed in CD4^+^ as well as in CD8^+^ T cells from adult peripheral blood but not in naïve T cells isolated from cord blood. Interestingly, ct‐CD45 levels in adult plasma were substantially higher (12.98 ± 5.04 ng/mL) than in plasma derived from umbilical cord blood (1.36 ± 0.28 ng/mL). Interestingly, there was a low number of clinically healthy individuals among our study population with distinctly higher levels of ct‐CD45, ranging between 140 and 190 ng/mL, suggesting a bimodal distribution of ct‐CD45 levels. These differences were not age related or sex related. At present, we can only speculate about reasons underlying this phenomenon. We cannot exclude that these individuals had asymptomatic infections at the time of the blood draw, leading to neutrophil activation and the rise of ct‐CD45 levels. However, other soluble mediators in serum, such as sICAM‐1, sL‐selectin, sP‐selectin, or sE‐selectin 29, 30 show distributions similar to ct‐CD45, suggesting that plasma levels of these molecules are variable in different individuals.

It is well‐known that the immune system of newborns differs in many molecular and functional aspects from the adult one 31. Our findings indicate that the immune‐regulatory function of ct‐CD45 may preferentially exist in adults but not at birth.

CD45 is an abundant cell surface receptor exclusively found on leukocytes 4. We have discovered that ct‐CD45 is released by neutrophils or monocytes upon activation‐induced proteolytic cleavage in vitro 5. Phagocytes have a relatively short life span. In view of the billions of phagocytes that die every day in our body it seems plausible to suggest that plasma ct‐CD45 is primarily phagocyte‐borne. Phagocytes from cord blood, in particular neutrophils, have impaired recruitment, chemotaxis, phagocytosis, bactericidal activity and oxidative burst formation capacity compared to cells from adult blood 32, 33, 34, 35. Therefore, it appears likely that newborns have reduced ct‐CD45 due to a less active phagocytic cell system. ct‐CD45 is not the only intracellular factor from phagocytes found in human plasma 27. Another example is high mobility group protein B1 (HMGB‐1), a factor with primary cellular functions in the nuclear compartment and the cytosol, which may be released from cells via necrosis or active secretion 27, 36. Extracellular HMGB‐1 has been shown to function as a “danger signal” leading to the activation of DCs and consequently promoting adaptive immunity 37. In contrast to HMGB‐1, ct‐CD45 might act as an inhibitory factor that helps to prevent exaggerated immune responses executed by T cells. The reutilization of intracellular proteins for alternative functions is an emerging theme in cell biology. Our findings suggest that this mechanism contributes to the immune‐regulatory characteristics of plasma.

T cells activated via CD3 or CD3/CD63 in the presence of ct‐CD45 showed a weak proliferative response, produced little cytokines and failed to upregulate typical activation markers. Costimulation via CD28 largely abrogated ct‐CD45 effects on T‐cell activation since neither proliferation nor blastogenesis were affected. However, also for CD3/CD28 stimulated cells, ct‐CD45 inhibited the production of IL‐2, IL‐4, IL‐17, and IL‐22, while other cytokines, including IFN‐γ, IL‐10, IL‐5, IL‐13, and TNF‐α were not affected, suggesting that T‐cell activation and function were differentially targeted. This phenomenon could be explained in part by the capability of CD28 to support IL‐2 independent growth 38, 39, which might sustain normal T‐cell growth even in the presence of low IL‐2. A study by Evavold and Allen suggested that the TCR may have the capacity of differential signaling, since an altered peptide ligand harboring a single amino acid substitution was capable of inducing IL‐4 production in the absence of T‐cell growth 40. Thus, it appears that ct‐CD45 and CD28 cosignals modulate these signals in different ways. As a net result, the cytokine release of CD3/CD28 stimulated is slightly polarized, while proliferation is unperturbed.

CD3 expression was not affected by ct‐CD45 and upregulation of CD69, which is preformed in the cytosol of T cells, was only slightly impaired. Most strikingly, ct‐CD45 inhibited lymphoblast formation for CD3/CD63 stimulated cells, while cells that were costimulated via CD28 had normal blastogenesis. Induction of blast formation is a typical sign of cells entering the cell cycle. Analysis of the cell‐cycle phases revealed that treatment of T cells with ct‐CD45 leads to an arrest during the G_0_/G_1_ transition. The expression levels of cyclins confirmed this observation. ct‐CD45 inhibited the induction of cyclin D2 and D3 as well as cyclin E1. Accordingly, *CDK2* and *CDK4* were also downregulated in T cells activated via CD3 or CD3/CD63 in the presence of ct‐CD45. In parallel, the cell‐cycle inhibitor *p27kip1* was induced in T cells upon activation in the presence of ct‐CD45. Again, this effect was not seen when T cells were stimulated via CD3/CD28.

ct‐CD45 may act on T cells via the modulation of a specific set of genes known to be involved in cell‐cyle regulation of T lymphocytes, including *KLF2*, a well‐defined regulator of T‐cell quiescence. Another, less defined gene that was induced in T cells by ct‐CD45 was *SLFN12*. Members of the SLFN family have been implicated in the control of cell growth and differentiation 22, 41, 42, 43, 44, 45 and have been identified in different vertebrate species 46. Nevertheless, humans and mice share only few one‐to‐one orthologues, which might be due to the rapid evolution of this gene family 46. *SLFN12* has no direct murine orthologue but is most similar to murine *Slfn2*, a damaging mutation of which has been shown to lead to a loss of immune cell quiescence 41. We have recently demonstrated that *SLFN12*—similar to *KLF2*—is highly expressed in human resting T cells from peripheral blood but is rapidly downregulated in these cells upon activation 16. *SLFN12* was diminished upon administration of exogenous IL‐2 and reduced T‐cell proliferation induced via CD3 or CD3/CD63 upon transgenic expression. This suggests a role for *SLFN12* in the regulation of immune cell quiescence in humans similar to the one ascribed to *Slfn2* in the mouse. Thus, *SLFN12* together with *KLF2* may contribute to the immunoregulation induced by ct‐CD45. However, since we do not possess global gene expression data at time points earlier than 12 h after T‐cell activation, we cannot exclude that other factors including, for example, Tob1 11, contribute to the initiation of the ct‐CD45‐induced inhibition of T‐cell function.

Recent studies have demonstrated that regulatory T and B cells play a central role in controlling T‐cell activation in vivo 47, 48. ct‐CD45 could be regarded as a soluble regulator of the steady state of T cells in plasma. Reduced levels of ct‐CD45 might facilitate the development of autoimmunity. Accordingly, we have observed that patients with RA or SLE, have less ct‐CD45 in their plasma than healthy controls. Interestingly, these patients also expressed lower levels of *SLFN12* in their T cells. In view of the fact, that SLFN12 is an interferon inducible gene 22 and that RA/SLE patients usually express a type I interferon signature 21, 49, 50, it is intriguing that T cells express less *SLFN12*. It is tempting to speculate that reduced levels of *SLFN12* are a consequence of lower levels of ct‐CD45 in RA/SLE patient plasma. Reasons underlying this phenomenon need to be clarified. One reason for the reduced presence of ct‐CD45 could be the increased formation of neutrophil extracellular traps reported in such patients 51, 52, which might retain ct‐CD45. Alternatively, other studies have demonstrated that neutrophils from RA patients are less apoptotic 53, 54, suggesting that ct‐CD45 might be released at lower levels. Another possibility could be depletion of soluble ct‐CD45 due to increased receptor expression on activated cells in these patients.

However, most of the patients of our study population were treated with anti‐inflammatory drugs (Table 1), suggesting that reduction of ct‐CD45 levels in RA and SLE patients might be due to certain immunosuppressive therapies rather than a result of a common disease mechanism. Indeed, our data seem to support this hypothesis, since we observed lower ct‐CD45 levels in RA patients receiving methotrexate and SLE patients treated with azathioprine, compared to patients with other treatments. Methotrexate and azathioprine are standard medication in the treatment of RA and SLE, respectively 55, 56. Methotrexate is a folic acid antagonist, which has diverse immunosuppressive function including, for example, the inhibition of phagocyte activation or the interference with TNF‐α production 24. Azathioprine is a prodrug, which mainly acts as a purine antagonist upon metabolic conversion, although its full mechanism of action is incompletely understood 57. Thus, given the potential of ct‐CD45 to serve as a prognostic and/or therapeutic marker, it would be interesting to analyze changes in ct‐CD45 levels of RA and SLE patients before and after treatment with immunosuppressive drugs such as methotrexate or azathioprine.

We have demonstrated that ct‐CD45 is binding to preactivated T cells, suggesting that there might be a specific receptor for ct‐CD45. However, the receptor for ct‐CD45 is still unknown, although CD26 has been described as a receptor for the cytoplasmic part of intact CD45 58. We have investigated if mAbs against CD26 can block binding and functional effects of ct‐CD45 in this study, but found no evidence that CD26 could be the receptor for ct‐CD45 (data not shown). CD45, and in particular its intracellular part, is known to interact with several proteins 4. We have started out to identify the receptor for ct‐CD45 on T cells by using a retroviral expression cloning approach 59 and discovered that cell surface expression of protein associated with Toll‐like receptor 4 (PRAT4A) 60 enables ct‐CD45 binding. However, binding studies using the Biacore system, indicated that ct‐CD45 and PRAT4A do not directly interact with each other, suggesting that PRAT4A might only be a cofactor for ct‐CD45 binding.

In summary, we demonstrate for the first time the physiological existence of ct‐CD45 in human plasma and show that it may be an extrinsic factor contributing to the maintenance of human T‐cell quiescence.

## Materials and methods

### Human serum and plasma samples

Cord‐blood samples were collected during healthy full‐term deliveries with approval from the Sankt Josef Hospital (Vienna, Austria) institutional review board and informed consent was obtained from the parents before birth. Plasma was generated from heparinized whole blood obtained from umbilical cord or peripheral blood from adults and processed within 6 h after collection. To obtain sera, blood was placed at 37°C for 1 h. The blood clot obtained was then spun at 500 g for 20 min and the serum removed from the upper layer.

Blood from patients and healthy controls was obtained from the “Biobank‐Project (EK Nr. 559/2005)” of the Department of Rheumatology (Medical University of Vienna) with ethical approval from the ethics committees of the Medical University of Vienna. RA and SLE patients used in this study were defined on the basis of seropositivity for autoantibodies. Disease activity in RA patients was measured according to CDAI and DAS28 scores 61. Further information on patients, including age, treatment, and clinical parameters, are listed in Table 1. Healthy controls were age matched and sex matched. For comparisons with RA patients, the mean age of controls was 54.7 ± 10.8 years. The mean age of the control population for SLE patients was 44.7 ± 19.7 years.

### Media, reagents, and chemicals

Cells were maintained in RPMI 1640 medium (Invitrogen, Paisley, UK) or in IMDM, supplemented with 2 mmol/L l‐glutamine, 100 U/mL penicillin, 100 μg/mL streptomycin (PAA Laboratories, Pasching, Austria), and 10% fetal calf serum (Life Technologies, Carlsbad, CA).

The following murine mAbs were raised in our laboratory: negative control antibody VIAP (against calf intestine alkaline phosphatase), 8–301 (against ct‐CD45), CD63‐11C9 (CD63), and VIT200 (CD45). Anti‐mouse CD45.2‐APC (clone 104) was obtained from eBioscience (Hatfield, UK). Goat anti‐mouse IgG and goat anti‐human IgG antibodies were purchased from Jackson Immunoresearch (Newmarket, UK). Oregon Green‐conjugated goat anti‐mouse IgG was acquired from Life Technologies. The OKT3 (CD3) antibody for T‐cell activation was obtained from Jansen‐Cilag (Vienna), anti‐CD28 was supplied by Caltag Laboratories (Burlingame, CA).

ct‐CD45‐Ig fusion proteins (full length, D1 and D2 constructs) were generated in our laboratory 5. CTLA4‐Ig (Belatacept) was used as a control Ig fusion protein and was purchased from Bristol Myers Squibb (New York, NY). Recombinant ct‐CD45 (aa 584–1281, from *Saccharomyces cerevisiae*) was purchased from Merck Millipore (Darmstadt, DE). Human recombinant IL‐2 was acquired from PeproTech (London, UK).

### Cell isolation

Buffy coats from healthy donors were purchased from the University Clinic for Blood Group Serology and Transfusion Medicine, Medical University of Vienna and the Austrian Red Cross (both, Vienna, Austria). Isolation of PBMCs was performed via standard density gradient centrifugation using Ficoll‐Paque Plus (GE Healthcare, Chalfont St. Giles, UK). T cells were isolated using the MACS system (Miltenyi Biotec, Bergisch Gladbach, Germany) as described 62. Purified T cells (total CD3^+^ T cells) were obtained via depletion of CD11b^+^, CD14^+^, CD16^+^, CD19^+^, CD33^+^, and MHC class II‐positive cells from total PBMCs. Cells were cryopreserved in RPMI 1640 supplied with 20% FCS and 10% DMSO using a Nalgene “Mr Frosty” 1°C freezing container (Thermo Fisher Scientific, Waltham, MA). Cells were stored in liquid N_2_ until use. After thawing at 37°C, cells were slowly diluted via drop‐wise addition of medium and washed twice to remove residual DMSO.

### Culture and activation of T cells

T cells were cultured at 2 × 10^5^ cells/well in 96‐well MAXISORP Nunc‐Immuno plates (Thermo Fisher Scientific) using full RPMI1640 growth medium. For ct‐CD45 depletion assays, RPMI1640 was supplemented with 20% human serum instead of 10% fetal calf serum. Activation of cells was performed via plate‐bound antibodies as described 63. To this end, plates were coated with 3 μg/mL Fc‐specific goat anti‐mouse IgG and 3 μg/mL goat anti‐human IgG (Jackson Immunoresearch Laboratories) overnight at 4°C, washed twice, and then incubated with 30 μg/mL of the respective fusion protein (ct‐CD45‐Ig or CTLA4‐Ig) plus anti‐CD3 (2 μg/mL). For costimulation, a combination of anti‐CD3/CD28 or anti‐CD3/CD63 mAb (2 μg/mL of each antibody) was coated. T‐cell proliferation was assessed via cellular incorporation of [methyl‐3H] thymidine (Perkin Elmer/New England Nuclear Corporation, Wellesley, MA). Cells were labeled with 0.05 mCi/well of [methyl‐3H] thymidine on day 3 of activation and cultured for another 18 h prior to harvesting. Detection was performed on a microplate scintillation counter (Topcount; Packard, Meriden, CT). Readings are displayed as counts per minute. All assays were performed in triplicates.

### ELISA

ct‐CD45 was detected and measured by a sandwich ELISA. Flat bottomed, 96 well ELISA plates (Corning Life Sciences, Tewskbury, MA) were coated with 100 μL of mAb 8–301 (5 μg/mL) overnight at 4°C. The plate was washed twice with a PBS‐Tween 0.5% solution. Blocking was performed by adding 200 μL of a 2% PBS‐BSA solution to each well and incubating again overnight at 4°C. After washing with PBS‐Tween, 100 μL of plasma or serum was added and incubated for 4 h at 4°C. Recombinant ct‐CD45 was used as positive control to generate a standard curve. The plate was washed then three times with PBS‐Tween. Afterwards, the rabbit clonal antibody E19‐G (DB Biotech, Kosice, SK) directed against a peptide (Glu1277‐Val1292) from the C‐terminal sequence of human CD45 was added (1:500 dilution) and incubated for 2 h at 4°C. After washing with PBS‐Tween three times, bound E19‐G antibody was detected with a goat‐anti‐rabbit antibody labeled with alkaline phosphatase (Jackson Immunoresearch). Unbound antibodies were removed by washing three times. Substrate buffer containing diethanolamine was added to analyze the bound antibodies colorimetrically using an ELISA reader (Bio‐Rad Laboratories, Hercules, CA). The detection limit of the ct‐CD45 ELISA is 0.8 ng/mL.

### Immunoprecipitation

For immunoprecipitation, 100 μg mAb 8–301 were loaded onto 7 × 10^7^ sheep anti–mouse IgG 2.8 μm Dynabeads (Thermo Fisher Scientific) as described elsewhere in detail 5, 64. After washing twice in PBS/0.01% BSA, the beads were incubated with serum or plasma for 24 h at 4°C on a rotator. Subsequently, the beads were washed with PBS, and bound protein was analyzed by flow cytometry.

### Gene expression profiling and analysis

Gene expression profiling and analysis was performed as described in detail elsewhere 65. Briefly, 1 × 10^7^ human peripheral blood T cells from five donors were stimulated for 12 and 24 h with plate‐bound CD3/CD28 and CD3/CD63 mAbs in the presence or absence of ct‐CD45. Cell samples were lysed in 500 μL of TRIzol (Life Technologies). Following RNA isolation, the aqueous phase containing the RNA was further purified using RNeasy MinElute Cleanup Kit (Qiagen). RNA integrity was confirmed on an Agilent 2100 Bioanalyzer (Agilent Technologies, Santa Clara, CA, USA). Hundred nanograms of total RNA was used for target preparation by 3′ IVT Express kit (Affymetrix, Santa Clara, CA, USA) according to the manufacturer's instructions. Hybridization cocktails were hybridized onto Human Genome U133 Plus 2.0 GeneChips (Affymetrix). Chips were read by a GeneChip scanner 3000 (Affymetrix). Microarray data were normalized and gene expression measures derived using the RMA algorithm and the Bioconductor package ‘Affy’ (http://www.bioconductor.org).

### Cloning of retroviral vectors

The SLFN12 coding sequence was obtained from a human monocyte‐derived dendritic cell cDNA expression library 59 by amplification using *SLFN12 fwd* 5’‐GCGCCCGGCCATTACGGCCGGACACTGCATAGCTGCTG‐3’ and *SLFN12 rev 5*’‐GCGCCCGGCCGAGGCGGCCCATCAGGTGAGCCTTCGAC‐3’ followed by digestion with *SfiI*. Gel‐purified DNA fragments were ligated into the retroviral expression vector pCJK2 66, sequenced and subcloned into the pMMP‐IRES‐EYFP 67 vector.

### Generation of retroviral supernatants

Phoenix gag‐pol cells were cultured in full IMDM growth medium. For transfection, cells were seeded into 100 mm dishes at 4 × 10^6^ cells per dish and transfected 24 h later. Fifteen micrograms expression vector per dish was transfected together with 5 μg of pMD‐gag‐pol and 10 μg of pMD‐RD114 67 via calcium phosphate precipitation. Retroviral supernatants were harvested on days 2 and 3 after transfection and concentrated by ultrafiltration.

### Retroviral transduction

Peripheral blood T cells or monocyte‐depleted PBMCs were stimulated at 1 × 10^6^ cells/well in 24‐well plates usingDynabeads Human T‐Activator CD3/CD28 (Life Technologies) and recombinant human IL‐2 (150 U/mL). Cells were harvested 2–3 days after activation, counted and resuspended in fresh medium at 1 × 10^7^ cells/mL. Hundred microliters of the cell suspension was mixed (1:10) with freshly harvested retroviral supernatants containing 8 μg/mL of polybrene and was centrifuged in 24 well plates at 800 g for 1–2 h at 30°C. After another 5 h of incubation at 37°C the transduction medium was exchanged to fresh growth medium containing IL‐2 (75 U/mL). Transduction efficiency was measured on day 4 after transduction using flow cytometry and was typically 60–80% as indicated by EYFP expression.

### Cytokine measurements

Supernatants from T‐cell activation experiments were harvested on day 4. Cytokines including IL‐2, IFN‐γ, IL‐4, IL‐5, IL‐13, IL‐17A, IL‐22, IL‐10, and TNF‐α were measured via the Luminex 100 System (R&D Systems Inc.) as described in the manufacturer's protocol. All measurements were performed in triplicates.

### Cell‐cycle analysis

Activated T cells were harvested on day 4 postactivation and were fixed in 70% ethanol at 4°C for 30 min. After washing, cells were stained with 50 μg/mL PI in the presence of 100 μg/mL RNAse A for 45 min at room temperature. DNA content was then measured on a BD FACSCalibur flow cytometer (BD Biosciences, San Jose, CA).

### RNA isolation and qPCR

Total RNA was prepared using peqGOLD TriFast reagent (peqLab, Erlangen, Germany). For isolation, 2 × 10^6^ cells/mL were lysed in 500 μL of TriFast reagent and isolated according to the manufacturer's protocol. One microgram of total RNA per sample was reverse transcribed using H‐Minus‐Reverse Transcriptase (Thermo Fisher) and oligo‐dT_18_ primers. Quantitative real‐time PCR (qPCR) was performed via the CFX 96 realtime‐PCR detection system (Bio‐Rad) using SYBR Green I (Bio‐Rad). Detection was performed according to the manufacturer's protocol. cDNA was amplified using a standard program (10 min at 95°C, 40 cycles of 15 s at 95°C/15 s at 60°C / 45 s at 72°C).

Primers for *CD3E* have been described before 68. Primers for *SLFN12* (cat. no. sc‐93836‐PR) and *KLF2* (cat. no. HP212088) were from Santa Cruz Biotechnology (Santa Cruz, CA) and Origene (Rockville, MD), respectively. Other primers were designed using the web‐based software Primer 3 plus (http://www.bioinformatics.nl/cgi‐bin/primer3plus/primer3plus.cgi) and are listed in Supporting Information Table 2.

### Flow cytometry

Flow cytometric analysis was performed as described previously 5. Briefly, for cell surface staining, 2 × 10^5^ cells per staining were incubated for 30 min at 4°C with either unconjugated mAb or APC‐conjugated mAb. After washing, secondary Oregon Green 488‐conjugated goat anti–mouse‐IgG (Life Technologies) was added to samples that had been stained with an unconjugated primary mAb. Intracellular stainings were performed by fixing cells for 20 min at room temperature using FIX solution followed by washing and permeabilization in the presence of primary antibody using PERM solution (both, An der Grub). After washing samples were incubated with secondary antibody in PERM solution and analyzed on a BD FACSCalibur flow cytometer (BD Biosciences).

### Statistical analysis

Data were analyzed using GraphPad Prism software (GraphPad Software, La Jolla, CA) applying Mann–Whitney *U* test or Kruskal–Wallis test with Dunn's posttest for multiple comparisons. *p* values: *p < 0.05; **p < 0.01; ***p < 0.001.

### Data access

The microarray data used in this study is accessible via Gene Expression Omnibus under the accession number GSE75723.

## Conflict of interest

The authors declare no financial or commercial conflict of interest.

**Abbreviations**
CDK: cyclin‐dependent kinase
ct‐CD45: cytoplasmic tail of CD45
KLF2: Krueppel‐like factor 2
PRAT4A: protein associated with Toll‐like receptor 4
RA: rheumatoid arthritis
SLE: systemic lupus erythematosus
SLFN12: Schlafen family member 12

## Supporting information

As a service to our authors and readers, this journal provides supporting information supplied by the authors. Such materials are peer reviewed and may be re‐organized for online delivery, but are not copy‐edited or typeset. Technical support issues arising from supporting information (other than missing files) should be addressed to the authors.

**Supporting Information Table 1**. Expression of selected regulators of T cell activation in the presence of ct‐CD45 determined via microarray analysis. Numbers denote log2 fold changes relative to untreated cells.

Peer review correspondence
