# Supplementary material for: The soluble cytoplasmic tail of CD45 (ct‐CD45) in human plasma contributes to keep T cells in a quiescent state
Source: Eur J Immunol. 2016 Oct 31;47(1):193–205. doi: 10.1002/eji.201646405 (PMC5244668; doi:10.1002/eji.201646405)
Supplement: Supplementary file 1 — Supporting Information Table 1. Expression of selected regulators of T cell activation in the presence of ct‐CD45 determined via microarray analysis. Numbers denote log2 fold changes relative to untreated cells. [file EJI-47-193-s001.pdf]

European Journal of Immunology

Supporting Information
for
DOI 10.1002/eji.201646405

Alexander Puck, Stefan Hopf, Madhura Modak, Otto Majdic, Petra Cejka, Stephan Blüml, Klaus Schmetterer, Catharina Arnold-Schrauf, Jens G. Gerwien, Klaus S. Frederiksen, Elisabeth Thell, Judith Leitner, Peter Steinberger, Regina Aigner, Maria Seyerl-Jiresch, Gerhard J. Zlabinger and Johannes Stöckl

The soluble cytoplasmic tail of CD45 (ct-CD45) in human plasma contributes to keep T cells in a quiescent state

© 2016 The Authors. *European Journal of Immunology* published by WILEY-VCH Verlag GmbH & Co. KGaA, Weinheim.

Supporting Information

Tables

Supporting Information Table 1. Expression of selected regulators of T cell activation in the presence of ct-CD45 determined via microarray analysis. Numbers denote log2 fold changes relative to untreated cells.

Symbol	CD3/CD28/ct-CD45 12 h	CD3/CD28/ct-CD45 24 h	CD3/CD63/ct-CD45 12 h	CD3/CD63/ct-CD45 24 h
CBLB	-0,1	-0,1	0,0	0,0
EGR2	-0,7	-0,8	-1,8	-1,9
EGR3	-0,6	-0,4	-1,9	-1,0
DGKA	0,3	0,1	0,1	0,4
TOB1	0,2	0,0	0,3	0,2
ITCH	0,0	-0,6	0,3	0,0
RNF128	0,0	0,0	-0,1	-0,1
SIRT1	0,1	0,3	0,0	0,1
KLF2	0,7	1,2	0,8	0,9
SLFN12	0,4	0,9	0,5	0,4
SLFN5	0,2	0,1	0,1	0,4
SLFN11	0,6	-0,7	0,3	0,1
SLFN13	0,1	0,0	-0,3	0,1

Supporting Information Table 2. Primers used for qPCR.

Target	Primer sequence
GAPDH_F	CGACCACTTTGTCAAGCTCA
GAPDH_R	AGGGGAGATTCAGTGTGGTG
CDK2_F	TTGTCAAGCTGCTGGATGTC
CDK2_R	TTTAAGGTCTCGGTGGAGGA
CDK4_F	TTCTGGTGACAAGTGGTGGA
CDK4_R	CTGGTCGGCTTCAGAGTTTC
CyclinD2_F	CTGGGGAAGTTGAAGTGGAA
CyclinD2_R	ATCATCGACGGTGGGTACAT
CyclinD3_F	AGACCTTTTTGGCCCTCTGT
CyclinD3_R	CCTGAGTGCAGCTTCGATCT
CyclinE1_F	AATGCGAGCAATTCTTCTGG
CyclinE1_R	CTGGTGCAACTTTGGAGGAT
p27kip_F	CCGGCTAACTCTGAGGACAC
p27kip_R	AGAAGAATCGTCGGTTGCAG

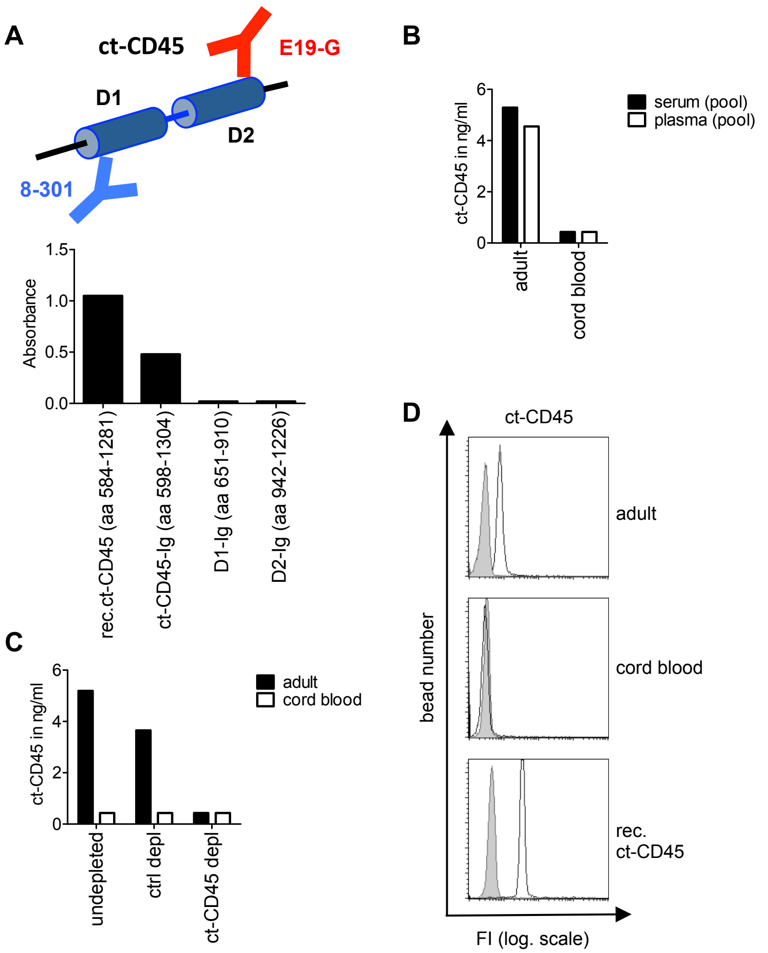

Supporting Information Figure 1. Detection and immunoprecipitation of ct-CD45. (A) Schematic depiction of the ct-CD45 domain composition and of the binding sites of the monoclonal antibodies used for the ELISA (above). Detection of recombinant ct-CD45 and ct-CD45-Ig as well as of truncated ct-CD45-Ig constructs, comprising only domain D1 or D2 via the ELISA system (below). Numbers in brackets indicate the region in the total CD45 amino acid (aa) sequence that is contained within the respective construct. (B) Comparison of ct-CD45 levels via ELISA in pooled plasma and pooled serum derived from adult and umbilical cord blood. (C) Analysis of ct-CD45 levels via ELISA in plasma treated with bead-coupled 8-301 mAb (ct-CD45 depl) or VIT200 mAb (ctrl depl) as a control. (D) Immunoprecipitation of recombinant or native ct-CD45 from plasma via bead-coupled 8-301 mAb. Beads were then stained using E19-G and secondary fluorochrome-conjugated antibody and analyzed via flow cytometry, gating on single beads.

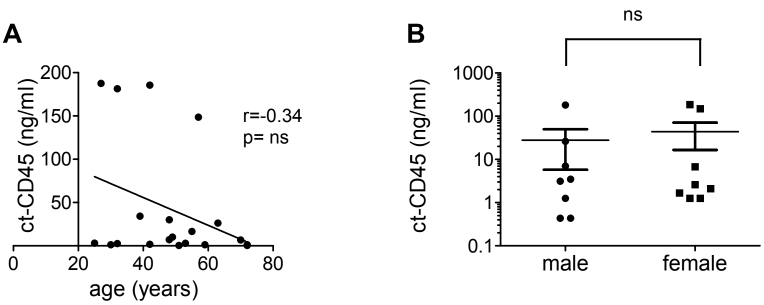

Supporting Information Figure 2. ct-CD45 levels do not correlate with age or sex. ct-CD45 levels were measured via ELISA in adult plasma and analyzed with respect to (A) age and (B) sex of the donors (male, n=8, mean age=46.1 ; female n=8, mean age=45.5). (A) Data was analyzed using linear regression and Pearson‘s r . r, Pearson correlation coefficient or (B) Mann-Whitney U test. P values: *, $p < 0.05$, not significant (ns) $p > 0.05$.

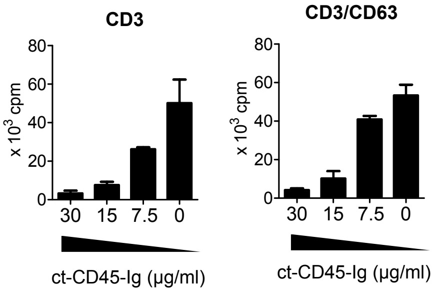

Supporting Information Figure 3. Titration of ct-CD45-Ig. Human T cells were stimulated via plate-bound CD3 or CD3/CD63 antibodies in the presence of graded amounts of ct-CD45-Ig. Concentrations indicated were used for coating of protein to 96 well plates. Proliferation was analyzed via thymidine incorporation. cpm, counts per minute. Data show mean ± SD and are representative of at least two independent experiments.

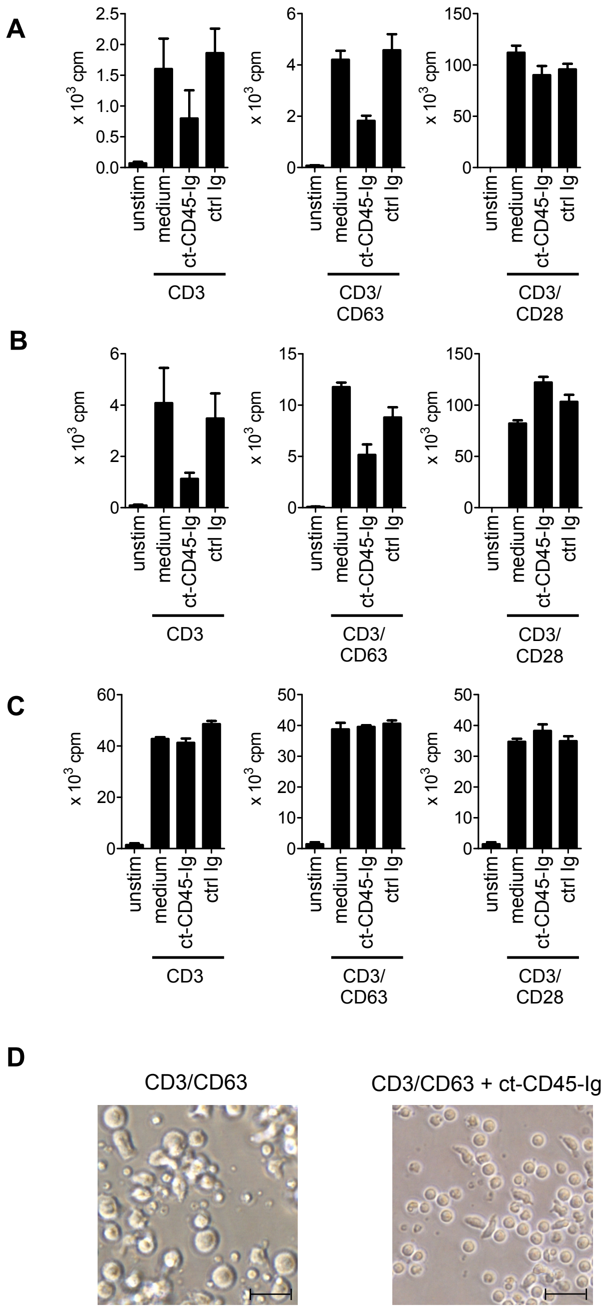

Supporting Information Figure 4. Effects of ct-CD45 on the proliferation of T cell subsets and cord blood T cells. T cells were stimulated via plate-bound CD3, CD3/CD63 or CD3/CD28 antibodies in the presence of medium alone, ct-CD45-Ig or ctrl Ig. Proliferation of (A) adult $CD3^+CD4^+$, (B) adult $CD3^+CD8^+$ and (C) cord blood $CD3^+$ T cells was analyzed via thymidine incorporation. cpm, counts per minute. unstim, unstimulated control. (D) Bright field micrograph of CD3/CD63-stimulated T cells that were activated for 3 days in the presence or absence of ct-CD45-Ig. Scale bar, 25 μm. (A-C) Data show mean ± SD and are representative of at least two independent experiments.

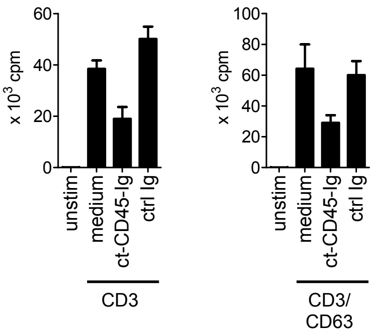

Supporting Information Figure 5. Effects of ct-CD45-Ig on the proliferation of freshly isolated $CD3^+$ T cells. T cells were stimulated via plate-bound CD3 or CD3/ CD63 antibodies in the presence of medium alone, ct-CD45-Ig or ctrl Ig. cpm, counts per minute. unstim, unstimulated control. Data show mean ± SD and are representative of at least three independent experiments.

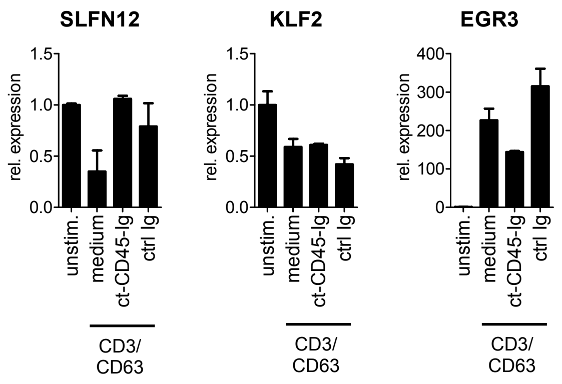

Supporting Information Figure 6. Expression of SLFN12, KLF2 and EGR3 6 hours after T cell activation in the presence of ct-CD45. Human CD3^{+} T cells were stimulated for 6 hours via plate-bound CD3/CD63 antibodies in the presence of medium alone, ct-CD45-Ig or ctrl Ig. mRNA levels of the indicated genes were analyzed via qPCR relative to *CD3E*. Data show mean ± SD and are representative of at least two independent experiments.

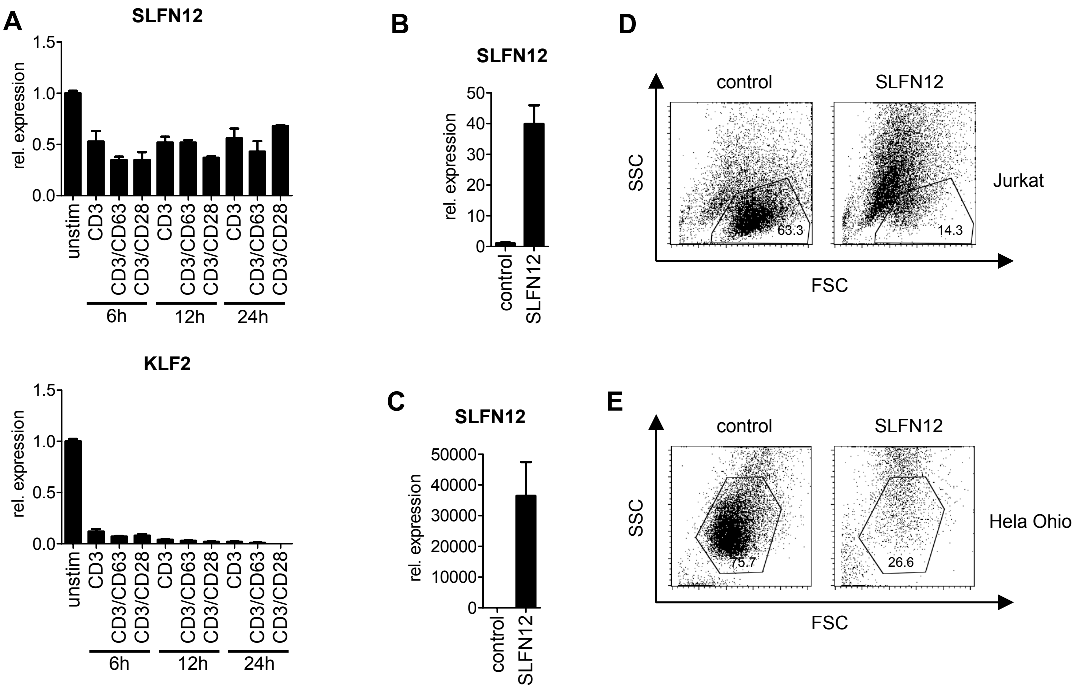

Supporting Information Figure 7. Regulation of SLFN12/KLF2 during T cell activation and SLFN12 overexpression experiments. (A) T cells were stimulated with plate-bound CD3, CD3/CD63 or CD3/CD28 antibodies over a 24 hours time course. Expression levels of *SLFN12* and *KLF2* were analyzed via qPCR using *CD3E* as a reference gene. (B) T cells were activated for 48 hours with Human T-Activator CD3/CD28 Dynabeads and recombinant human IL-2 (150 U/ml) for 2-3 days and were retrovirally transduced with *SLFN12* and control vectors. Cells were then expanded in IL-2 (75 U/ml). *SLFN12* mRNA expression was determined via qPCR on day 10 after gene transduction. (C) *SLFN12* mRNA expression in Jurkat cells transduced with *SLFN12* or empty control vector on day 3 after gene transduction. (D) FSC/SSC analysis of Jurkat cells on day 3 post transduction. (E) FSC/SSC analysis of *SLFN12*-transduced Hela Ohio cells on day 3 after gene transduction. (D, E) Numbers given indicate percentages of live cells in the gate. (A-C) Data are expressed as mean ± SD and (A-E) are representative of at least 3 independent experiments.

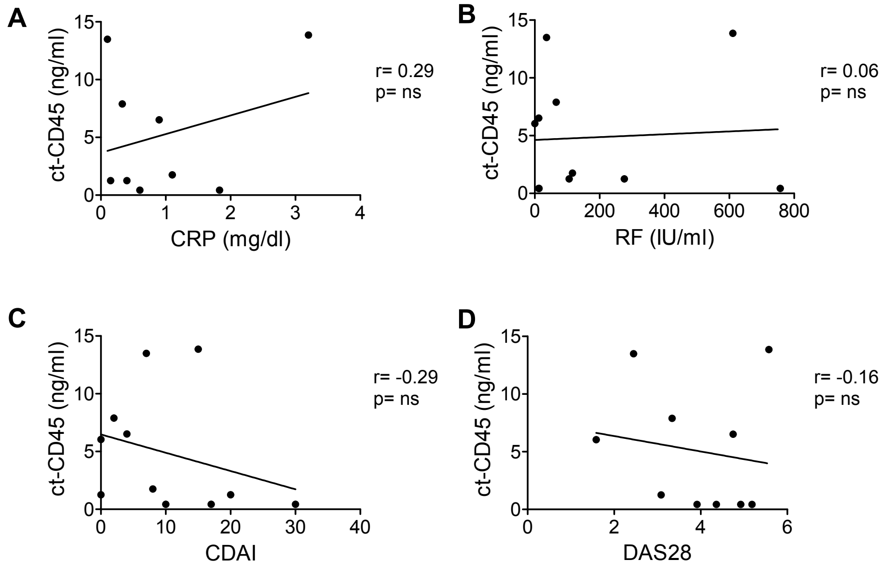

Supporting Information Figure 8. ct-CD45 levels do not correlate with clinical parameters of RA patients. ct-CD45 was measured via ELISA in RA patient serum and was correlated with (A) c-reactive protein (CRP) and (B) rheuma factor (RF) levels and with the disease activity scores (C) CDAI and (D) DAS28. Data was analyzed using linear regression and Pearson‘s r . r, Pearson correlation coefficient. P values: *, $p < 0.05$, not significant (ns), $p > 0.05$.

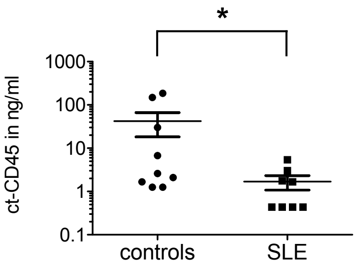

Supporting Information Figure 9. ct-CD45 levels are reduced in patients diagnosed with systemic lupus erythematosus (SLE). ct-CD45 was measured via ELISA in plasma derived from SLE patients (n=8) and compared to matched controls (n=9). Mean ± SEM are given. P values: *, $p < 0.05$. Mann-Whitney U test.

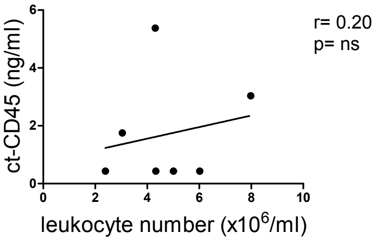

Supporting Information Figure 10. ct-CD45 levels do not correlate with leukocyte numbers of SLE patients. Ct-CD45 levels were measured via ELISA in sera of SLE patients and correlated with leukocyte numbers determined at the time of the blood draw. Linear regression and Pearson's r were calculated. r, Pearson correlation coefficient. P values: *, $p < 0.05$, not significant (ns), $p > 0.05$.

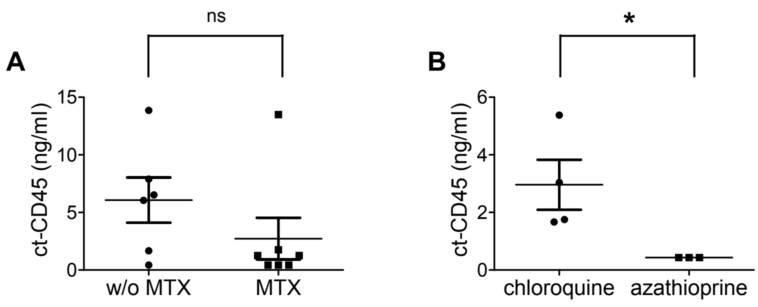

Supporting Information Figure 11. Differential Effects of immunosuppressive treatment on ct-CD45 levels of RA and SLE patients. Ct-CD45 levels were analyzed via ELISA in sera obtained from (A) RA patients receiving treatment that includes methotrexate (MTX) or that received treatment other than MTX (w/o MTX) and (B) SLE patients that were either treated with chloroquine or azathioprine. Mean ± SEM are given. Mann-Whitney U test. P values: *, $p < 0.05$.
