# Supplementary material for: The soluble cytoplasmic tail of CD45 (ct‐CD45) in human plasma contributes to keep T cells in a quiescent state
Source: Eur J Immunol. 2016 Oct 31;47(1):193–205. doi: 10.1002/eji.201646405 (PMC5244668; doi:10.1002/eji.201646405)
Supplement: Supplementary file 2 — Peer review correspondence [file EJI-47-193-s002.pdf]

European Journal of Immunology

The soluble cytoplasmic tail of CD45 (ct-CD45) in human plasma contributes to keep T cells in a quiescent state

Alexander Puck, Stefan Hopf, Madhura Modak, Otto Majdic, Petra Cejka, Stephan Blüml, Klaus Schmetterer, Catharina Arnold-Schrauf, Jens G. Gerwien, Klaus S. Frederiksen, Elisabeth Thell, Judith Leitner, Peter Steinberger, Regina Aigner, Maria Seyerl-Jiresch, Gerhard J. Zlabinger and Johannes Stöckl

Correspondence: Dr. Johannes Stockl, Institute of Immunology, Center for Pathophysiology, Infectiology and Immunology, Medical University of Vienna, Vienna, Austria

---

Review Timeline:	Submission date:	10-Mar-2016
	First Editorial decision:	12-Apr-2016
	First revision received:	07-Jul-2016
	Second Editorial decision:	28-Jul-2016
	Second revision received:	23-Aug-2016
	Third Editorial decision:	23-Sep-2016
	Accepted:	05-Oct-2016

Handling Executive Committee member: Prof. Francesco Annunziato

Please note that the correspondence below does not include the standard editorial instructions regarding preparation and submission of revised manuscripts, only the scientific revisions requested and addressed.

First Editorial Decision - 12-Apr-2016

Dear Dr. Stockl,

Manuscript ID eji.201646405 entitled "The soluble cytoplasmic tail of CD45 (ct-CD45) in human plasma contributes to keep T cells in a quiescent state" which you submitted to the European Journal of Immunology has been reviewed. The comments of the referees are included at the bottom of this letter.

A revised version of your manuscript that takes into account the comments of the referees will be reconsidered for publication. Should you disagree with any of the referees' concerns, you should address this in your point-by-point response and provide solid scientific reasons for why you will not make the requested changes. Please note that you do not need to change this manuscript into a Short Communication article, as suggested by referee #2.

You should also pay close attention to the editorial comments included below. **In particular, please edit your figure legends to follow Journal standards as outlined in the editorial comments. Failure to do this will result in delays in the re-review process.**

Please note that submitting a revision of your manuscript does not guarantee eventual acceptance, and that your revision will be re-reviewed by the referees before a decision is rendered.

If the revision of the paper is expected to take more than three months, please inform the editorial office. Revisions taking longer than six months may be assessed by new referee(s) to ensure the relevance and timeliness of the data.

Once again, thank you for submitting your manuscript to European Journal of Immunology and we look forward to receiving your revision.

Yours sincerely,
Laura Soto Vazquez

On behalf of
Prof. Francesco Annunziato

Editorial Office
European Journal of Immunology
e-mail: ejied@wiley.com
www.eji-journal.eu

****

European Journal of Immunology

Reviewer: 1

Comments to the Author

This is an interesting paper in which the Authors suggest that the soluble tail of CD45, ct-CD45, in plasma has a crucial role in controlling T cell activation and function. These Authors described a while ago that ct-CD45 was able to inhibit T cell proliferation (Blood 2008, 112:1240-1248). Here, the Authors make an attempt to further exploit the meaning of the previous data. First, they measure the presence of ct-CD45 in adult blood and compare these values to the ones present in cord blood. Actually, looking at these values, it seems that in the adult population there is a sort of bimodal distribution of ct-Cd45. Is that correct? If that is correct, the Authors should discuss these findings. The effects of ct-CD45 on ILs production (Fig.3) are not of univocal interpretation and may be worth a more detailed discussion. Assuming that for costimulation the key element is CD28, the data are quite different if you look at gamma-IFN, IL-13, TNFalpha and so on. How do the Authors explain the different effects of ct-CD45 on ILs production? Looking at these data, I got the view that ct-CD45 impairs T cell activation but affects T cell function in different ways. Is that due to the importance of costimulation in activation of functional pattern independently from cell proliferation? This issue may be worth to be further exploited. The expression of quiescence genes is very intriguing. However, I think that this issue needs to be more detailed. Looking at supporting data, it is evident that the expression of the genes changes over time. I understand that the Authors chose to investigate the ones that were found more expressed at 24 hours; it may be worth to look at a more comprehensive analysis. I see that TOB1 goes up at 12 hours and the role of this gene in controlling T cell activation is known. Therefore, on the basis of data obtained looking at ILs production, it is likely that we may see that there is a different efficiency of this given pattern depending on T cell subsets. Moreover, it may be worth to look at earlier time points, it may be that the gene machinery that goes up within the first 4-6 hours can tell us a more clear story. Finally, the data generated on patients affected by SLE or AR are difficult to understand. There are too many variables, starting from the fact that SLE and AR are heterogeneous diseases. Also here, SLFN12 expression has sort of bimodal distribution in both patients and controls. I was wondering if there is any difference comparing not the patients and the controls but SLE and AR patients.

Reviewer: 2

European Journal of Immunology

Comments to the Author

The manuscript by Puck et al. investigated the functional relevance of soluble cytoplasmic tail of CD45 on T cell activation. Their data clearly demonstrate the presence of ct-CD45 in adult plasma/serum and propose a regulatory function on T cell activation in particular in the case TCR stimulation is weak.

General comment

Although I am not an English native speaker myself, my impression is that overall the language should be (definitely) improved.

Other comments

• In the material & method section, it is written that cells were either directly used or freezed in liquid nitrogen (and the thawed for usage). Since the use of thawed cells can have a profound effect in particular in functional assays, it would be good to give the information where fresh and where frozen and thawed cells have been used. It may be even essential to demonstrate for the key experiments of the manuscript that there is no different outcome in such experiments using the different cell sources.
• Figure 1B/C/D: The authors clearly demonstrated that adult blood has more ct-CD45. One good addition in this respect would be to analyze blood from different age groups on the basis of the hypothesis that ct-CD45 might increase with (healthy aging) and correlate with other published signatures of “inflammaging” (eg IL6). In addition, experiments titrating CD3 and CD63/CD28 would give this part more impact
• The relative proliferation (fold of mock) is not very clear, what is “mock” is not defined or explained in neither the material & method section nor in the figure legend.
• The reason for choosing CTLA4 as a control fusion protein is not explained in the text.
• Figure 3B: information for labeling in histograms regarding the color code for lines is provided in the figure legend but it would be nice to have it in the figure as well.
• Is there a reason using different reference genes for qPCR analysis for Figure 4 and 7?
• The authors tried to connect the soluble ct-CD45 levels with RA/SLE patients via SLNF12 expression levels, however, the connection is very weak and using MxA as a reference gene to define the type 1 interferon signature completely failed. In addition the authors should provide more information on the patients; e.g. disease duration, disease activity and treatment. Moreover, information on the age of the

analyzed probands (healthy and patients) should be given. As compared to healthy probands and patients only a very low number (5) of SLE patients have been analyzed. The question arises whether this number is too low and in addition how the authors age matched the usually younger SLE patients with on average older RA patients.

• While an IFNalpha signature has been documented very well for SLE patients I was not aware that this is also true for RA patients. In this respect maybe the SLE patients anyway should be omitted from the analyses.

• Without any further data or analyses (e.g. analyses during successful (although only symptomatic) therapies with biologicals the ct 45 data from the patients read very preliminary. Either this section should be improved or skipped.

My overall feeling is that the manuscript as it stands should be rewritten and improved with the suggested data sets. The authors might consider presenting the key message of the paper rather as short communication than as a regular article.

First Revision – authors' response
07-Jul-2016

Comments of Reviewer #1 to the authors:

This is an interesting paper in which the Authors suggest that the soluble tail of CD45, ct-CD45, in plasma has a crucial role in controlling T cell activation and function. These Authors described a while ago that ct-CD45 was able to inhibit T cell proliferation (Blood 2008, 112:1240-1248). Here, the Authors make an attempt to further exploit the meaning of the previous data. First, they measure the presence of ct-CD45 in adult blood and compare these values to the ones present in cord blood. Actually, looking at these values, it seems that in the adult population there is a sort of bimodal distribution of ct-Cd45. Is that correct? If that is correct, the Authors should discuss these findings. The effects of ct-CD45 on ILs production (Fig.3) are not of univocal interpretation and may be worth a more detailed discussion. Assuming that for costimulation the key element is CD28, the data are quite different if you look at gamma-IFN, IL-13, TNFalpha and so on. How do the Authors explain the different effects of ct-CD45 on ILs production? Looking at these data, I got the view that ct-CD45 impairs T cell activation but affects T cell function in different ways. Is that due to the importance of costimulation in activation of functional pattern independently from cell proliferation? This issue may be worth to be further exploited. The expression of quiescence genes is very intriguing. However, I think that this issue needs to be more

detailed. Looking at supporting data, it is evident that the expression of the genes changes over time. I understand that the Authors chose to investigate the ones that were found more expressed at 24 hours; it may be worth to look at a more comprehensive analysis. I see that TOB1 goes up at 12 hours and the role of this gene in controlling T cell activation is known. Therefore, on the basis of data obtained looking at ILs production, it is likely that we may see that there is a different efficiency of this given pattern depending on T cell subsets. Moreover, it may be worth to look at earlier time points, it may be that the gene machinery that goes up within the first 4-6 hours can tell us a more clear story. Finally, the data generated on patients affected by SLE or AR are difficult to understand. There are too many variables, starting from the fact that SLE and AR are heterogeneous diseases. Also here, SLFN12 expression has sort of bimodal distribution in both patients and controls. I was wondering if there is any difference comparing not the patients and the controls but SLE and AR patients.

Authors' response to Reviewer #1:

We thank the reviewer for the constructive criticism of the data presented in our manuscript.

One of the reviewer's questions concerns a potential bimodal distribution of ct-CD45 in the adult population. Among our samples we do indeed find a small number of clinically healthy individuals with high levels of ct-CD45 in the range of 140-190 ng/ml, while the general mean appears to be much lower (12.98 ± 5.04 ng/ml). We have analyzed whether ct-CD45 levels in general could be age- or sex-related, but found no statistically significant correlation (please see our new Supporting Information Figure 2). At present, we can only speculate about reasons underlying this phenomenon. It might be that these individuals had asymptomatic infections at the time of the blood draw, leading to neutrophil activation and rise of ct-CD45 levels. However, other soluble mediators in serum, such as sICAM-1, sL-selectin, sP-selectin, sE-selectin (Hasegawa et al., PLOS One, 2014; Klok et al., Br. J. Ophthalmol., 1994) show distributions similar to the one of ct-CD45, suggesting that plasma levels of these molecules are variable in different individuals (please also see discussion on page 12, 1st paragraph).

Proliferation and cytokine production were affected by ct-CD45 in CD3 and CD3/CD63-stimulated cells, whereas CD28-costimulation appears to lead to differential effects. While proliferation was normal, cytokine expression was polarized since the production of IL-2, IL-4, IL-17 and IL-22 was inhibited, while other cytokines, including IFN-γ, IL-10, IL-5, IL-13 and TNF-α were not or only little affected. This might be explained in part by the capability of CD28 to support IL-2 independent T cell growth (Boulgouris et al., J Immunol, 1999; Colombetti et al, J Immunol, 2006), thus leading to normal T cell growth, despite low IL-2. Cytokine expression may be uncoupled from T cell proliferation, depending on the TCR signaling strength (Evavold and Allen, Science, 1991), which could explain some of the differential effects that were observed. As requested, we have included a more detailed discussion on this topic in the Discussion section of our revised manuscript (see page 13, 2nd paragraph).

European Journal of Immunology

Regarding the quiescence gene expression signature, we focused on the two genes most prominently expressed at the examined time points (12h, 24h). Tob1 was not strongly regulated at these endpoints. We have analyzed the expression of SLFN12, KLF2 and EGR3 via qPCR also at an earlier time point (6 hours) (please see our new Supporting Information Figure 6 and page 8, 2nd paragraph) and found that while SLFN12 was already slightly up-regulated, KLF2 expression was not different to controls. As a control, the early growth response gene EGR3 was down-regulated in ct-CD45-treated T cells. Its early regulation, as well as IL-2 mediated downregulation suggests that SLFN12 could play an important role in ct-CD45 effects. However, since we do not have global gene expression data for these early time points, we cannot rule out that other quiescence genes such as Tob1 were strongly regulated at earlier time points, which might hint at a concerted action of several factors. To acknowledge these limitations, we have commented on this in the discussion section of our manuscript (see page 15, 1st paragraph).

Comments of Reviewer #2 to the authors:

The manuscript by Puck et al. investigated the functional relevance of soluble cytoplasmic tail of CD45 on T cell activation. Their data clearly demonstrate the presence of ct-CD45 in adult plasma/serum and propose a regulatory function on T cell activation in particular in the case TCR stimulation is weak.

General comment

Although I am not an English native speaker myself, my impression is that overall the language should be (definitely) improved.

Other comments

• In the material & method section, it is written that cells were either directly used or freezed in liquid nitrogen (and the thawed for usage). Since the use of thawed cells can have a profound effect in particular in functional assays, it would be good to give the information where fresh and where frozen and thawed cells have been used. It may be even essential to demonstrate for the key experiments of the manuscript that there is no different outcome in such experiments using the different cell sources.

• Figure 1B/C/D: The authors clearly demonstrated that adult blood has more ct-CD45. One good addition in this respect would be to analyze blood from different age groups on the basis of the hypothesis that ct-CD45 might increase with (healthy aging) and correlate with other published signatures of “inflammaging” (eg IL6). In addition, experiments titrating CD3 and CD63/CD28 would give this part more impact

European Journal of Immunology

• The relative proliferation (fold of mock) is not very clear, what is "mock" is not defined or explained in neither the material & method section nor in the figure legend.

• The reason for choosing CTLA4 as a control fusion protein is not explained in the text.

• Figure 3B: information for labeling in histograms regarding the color code for lines is provided in the figure legend but it would be nice to have it in the figure as well.

• Is there a reason using different reference genes for qPCR analysis for Figure 4 and 7?

• The authors tried to connect the soluble ct-CD45 levels with RA/SLE patients via SLNF12 expression levels, however, the connection is very weak and using MxA as a reference gene to define the type 1 interferon signature completely failed. In addition the authors should provide more information on the patients; e.g. disease duration, disease activity and treatment. Moreover, information on the age of the analyzed probands (healthy and patients) should be given. As compared to healthy probands and patients only a very low number (5) of SLE patients have been analyzed. The question arises whether this number is too low and in addition how the authors age matched the usually younger SLE patients with on average older RA patients.

• While an IFNalpha signature has been documented very well for SLE patients I was not aware that this is also true for RA patients. In this respect maybe the SLE patients anyway should be omitted from the analyses.

• Without any further data or analyses (e.g. analyses during successful (although only symptomatic) therapies with biologicals the ct 45 data from the patients read very preliminary. Either this section should be improved or skipped.

My overall feeling is that the manuscript as it stands should be rewritten and improved with the suggested data sets. The authors might consider presenting the key message of the paper rather as short communication than as a regular article.

Authors' response to Reviewer #2:

We thank the reviewer for the comments and suggestions concerning our manuscript.

• The description that was given in the method section stating that either fresh or cryopreserved cells were used for experiments is inaccurate. All data shown in the manuscript stems from experiments using only cryopreserved cells that were derived from buffy coats. The prior statement solely refers to the fact that fresh cells were used in some RNAi experiments involving nucleofection since we wanted to assess whether there were differences in transfection and knockdown efficacy to cryopreserved cells. In these experiments, we found no major differences in the proliferation of cryopreserved (and thawed) T cells compared to freshly isolated cells and have not found differences regarding the inhibitory effect of ct-CD45-Ig (please see our new Supporting Information Figure 5). This is in line with reports in the

literature, which also indicate that cryopreservation has no major impact on CD4+ or CD8+ T cell subset composition (Galeano et al., Immunol Cell Biol, 2016, Weinberg et al., Clin Vaccine Immunol, 2009). However, the RNAi experiments failed due to technical reasons (no detectable knockdown) (see also comment on page 8, end of 2nd paragraph) and were finally not included in the paper. In order to avoid further misconceptions on this issue we have decided to change the respective description in the methods section (see "Cell isolation" page 19/20).

• The term "rel. prolif. to mock" in the y axis label of Figure 1 (B-D) should actually read "rel. prolif. to undepleted". The data is thus displayed relative to the proliferation of cells cultured in serum not treated with beads or antibody (termed "undepleted" on the x axis label). We apologize for this mistake and have corrected it in the figure and the figure legend.

• CTLA4-Ig has been described to inhibit T cell activation by interfering with the T cell-APC interaction, but does not act directly on T cells (Mayer et al., Int. Immunopharmacol., 2013; Rochman et al., PloS ONE, 2015). CTLA-Ig thus serves as a negative control and as a means to confirm T cell purity in an APC-free culture system. Our results confirm that purified T cells activated in the presence of CTLA4-Ig show no differences in their proliferation to cells activated without any fusion protein (Fig. 2A), while T cells activated in the presence of ct-CD45-Ig were inhibited.

• Histogram legends were included in Figure 3B as requested.

• Different reference genes were used on the basis of lower inter-donor variability of B2M mRNA expression compared to CD3E or GAPDH. The latter were found to be stable in activated T cells of the same donor during time course kinetics, but were less suitable for comparing T cells of different donors.

• We have demonstrated lower levels of ct-CD45 and of SLFN12 mRNA expression in RA/SLE patients compared to healthy controls. In this context, the reviewer raised concerns regarding the suitability of MxA as a control gene. MxA was chosen since it is a classical interferon-stimulated gene and shows similar induction kinetics as SLFN12 in human immune cells stimulated via IFN-α (Puck et al, Results Immunol., 2015). Intriguingly, we found that SLFN12 and MxA were not co-regulated in this setting, indicating that other factors were interfering with the expression of these two genes. Both, SLE and RA have been associated with type I interferons, but it is important to stress that a strong type I interferon signature is typically only found within a subset of RA patients (van der Pouw Kraan, Ann. Rheum. Dis., 2008). Since only three out of 20 patients show high MxA levels our data appear consistent with the literature. Another aspect, that has to be considered, is that the majority of patients were receiving drugs like TNF-inhibitors or methotrexate (please see our new Table 1). Thus, we cannot rule out treatment-related effects on ct-CD45 or SLFN12 and MxA expression. We attempted to statistically analyze possible effects resulting from treatment, but found that such an analysis was not feasible, since several patients received more than one treatment and only two patients were not receiving any treatment. Methotrexate treatment may also directly affect neutrophil function, which we discuss in our revised manuscript (page 16, 2nd paragraph). We agree with the reviewer that a study analyzing patients before and after treatment would be an ideal situation, however, this would exceed the scope of this

explorative study. Nevertheless, given the potential of ct-CD45 to serve as a prognostic or therapeutic marker, we consider the reviewer's comment as an interesting suggestion for further studies.

Since the reviewer raised concerns regarding the low number (n=5) of SLE patients involved, we increased this number and have analyzed sera of three more patients. Additionally, SLE patient data was moved to the Supporting Information (please see new Supporting Information Figure 9). Since it was not possible to obtain more RNA samples from SLE patients, we have decided not to include these results in the manuscript. As requested, we have also included further information (age, treatment etc.) on the patients and the controls within the methods section (page 18, 2nd paragraph) and Table 1. We have also analyzed whether ct-CD45 is linked to factors such as age, sex or inflammatory clinical parameters such as C-reactive protein (CRP) or rheuma factor (RF), but found no statistically significant correlation (please see new Supporting Information Figure 2 and 8).

Thus, although ct-CD45 appears to be reduced in RA and SLE patients, the exogenous and endogenous drivers interfering with ct-CD45 generation remain unknown to us. We hope that future studies may help to solve this issue.

We have taken the reviewer's concerns regarding language seriously and have sent it for proofreading. Corrections were implemented accordingly. The format of the manuscript was not changed to a "short communication" style according to the editor's comment on this aspect.

Second Editorial Decision
28-Jul-2016

Dear Dr. Stockl,

Thank you for submitting your revised manuscript ID eji.201646405.R1 entitled "The soluble cytoplasmic tail of CD45 (ct-CD45) in human plasma contributes to keep T cells in a quiescent state" to the European Journal of Immunology. Your manuscript has been re-reviewed and the comments of the referees are included at the bottom of this letter.

Although the referees have recommended publication, you will see that Referee 2 feels that a crucial measurement should be included. The Editor-in-chief agrees and I therefore I invite you to respond to the comments of Referee 2 and revise your manuscript accordingly.

You should also pay close attention to the editorial comments included below. *In particular, please edit your figure legends to follow Journal standards as outlined in the editorial comments. Failure to do this will result in delays in the re-review process.*

If the revision of the paper is expected to take more than three months, please inform the editorial office. Revisions taking longer than six months may be assessed by new referee(s) to ensure the relevance and timeliness of the data.

Once again, thank you for submitting your manuscript to European Journal of Immunology. We look forward to receiving your revision.

Yours sincerely,
Karen Chu

on behalf of Prof. Francesco Annunziato

Dr. Karen Chu
Editorial Office
European Journal of Immunology
e-mail: ejied@wiley.com
www.eji-journal.eu

****

Reviewer: 1

Comments to the Author
I think the Authors covered all the issues raised by this reviewer

Reviewer: 2

Comments to the Author
The authors have commented almost all remarks. However, still the patient data (the obtained results are not really strengthening the main findings or conclusions) remains weak. The shown data (e.g. lower ct-CD45 levels in RA and now more SLE patients) stands a little bit alone, since no further analysis is performed to explain these findings. In particular this is puzzling since both diseases represent autoinflammatory diseases of great heterogeneity and the selected patients in this study are characterized

by great heterogeneity (with respect to disease duration and treatments).

A) The authors write:
To test whether ct-CD45 levels were directly linked to clinical parameters of RA patients, we performed linear regression analysis with C-reactive protein (CRP) (Supporting Information Fig. 8A), and rheuma factor (RF) (Supporting Information Fig. 8B), but found no statistically significant correlation.

However, it is my opinion that CRP is definitely not a very good marker to be used as clinical parameter and also RF values do not correlate with e.g. disease activity in RA patients (while it can be of course used to subgroup RA patients). I am therefore wondering whether DAS data (Disease Activity Score for rheumatoid arthritis) is available for the selected patients. In a similar manner the SLEDAI represents a quite accepted score to classify disease activity in SLE patients.

Just to show that now 9 SLE patients are characterized by an apparently strong reduction of ct-CD45 is surprising. Can the authors correlate the obtained values also with the degree of leukopenia, a classical finding in SLE patients? Usually, patients with active disease have 10-100fold less reduced leukocyte counts. In this case it would be interesting whether soluble ct-CD45 would correlate with that.

As stated, as it stands it is my opinion that the shown patient data rather dilute the message of this paper, since solely demonstrating that in diseases different such as RA and SLE in general ct-CD45 is reduced remains an obscure finding. A similar pathogenic mechanism should not be involved in these different diseases, leaving the question open whether then the findings might be a result of immunsuppressive therapies in general. The authors are correct that adding new data from early diagnosis patients would take too long and is therefore not an appropriate solution. I therefore would suggest that the authors add data (that should be already there) that more specifically tries to clarify how ct-CD45 is associated to disease activity. One could foresee two scenarios: either reduced ct-CD45 levels are associated with high disease activity (could then indeed point out to a pathogenic mechanism or the opposite (maybe then ct-CD45 depend on the degree of immune suppression).

At least that would give the authors the opportunity to state more solid hypotheses. Without such data the shown reductions of ct-CD45 just remain enigmatic. On my opinion the shown patient data in the current version of the manuscript could also be omitted (if no additional disease activity score data are added).

Second Revision – authors' response
23-Aug-2016

Response to Reviewer 2

European Journal of Immunology

We thank the reviewer for the interest in our study and the critical evaluation of our manuscript.

We have followed your proposal to compare ct-CD45 levels with Disease Activity Scores for RA patients (CDAI and DAS28) and leukocyte counts for SLE patients, since SLEDAI scoring is not available. The results of this correlation are presented in our new Supplementary Figure 8C, 8D and Supplementary Figure 10, respectively, and demonstrate that there is no significant direct correlation between disease activity scores and ct-CD45 levels.

We have therefore also analyzed a potential link between ct-CD45 levels in our patient cohort and treatment strategy. Results presented in our new Supplementary Figure 11 show that RA patients treated with methotrexate have lower levels than patients treated with medications without methotrexate. This correlation is however not significant. Interestingly, when we analyzed the treatments of the SLE patients we observed that patients treated with azathioprine had no detectable ct-CD45 in the serum, compared to patients treated with chloroquine. Methotrexate and azathioprine are potent immuno-suppressive agents, which act via multiple mechanisms. Thus, we conclude from our data that the observed lower levels of ct-CD45 in RA and SLE patients may rather be the result of certain immune-suppressive treatment strategies than due to common pathogenic mechanisms in autoimmune patients.

This information is now also given in the Results section on page 10, last paragraph, and in the Discussion on page 17, 2nd paragraph.

Third Editorial Decision

23-Sep-2016

Dear Dr. Stockl,

It is a pleasure to provisionally accept your manuscript entitled "The soluble cytoplasmic tail of CD45 (ct-CD45) in human plasma contributes to keep T cells in a quiescent state" for publication in the European Journal of Immunology. For final acceptance, please follow the instructions below and return the requested items as soon as possible as we cannot process your manuscript further until all items listed below are dealt with.

Please note that EJI articles are now published online a few days after final acceptance (see Accepted

European Journal of Immunology

Articles: http://onlinelibrary.wiley.com/journal/10.1002/(ISSN)1521-4141/accepted). The files used for the Accepted Articles are the final files and information supplied by you in Manuscript Central. You should therefore check that all the information (including author names) is correct as changes will NOT be permitted until the proofs stage.

We look forward to hearing from you and thank you for submitting your manuscript to the European Journal of Immunology.

Yours sincerely,
Nadja Bakocevic

on behalf of
Prof. Francesco Annunziato

Dr. Nadja Bakocevic
Editorial Office
European Journal of Immunology
e-mail: ejied@wiley.com
www.eji-journal.eu
